# Neuroanatomical tract-tracing techniques that did go viral

**DOI:** 10.1007/s00429-020-02041-6

**Published:** 2020-02-15

**Authors:** Jose L. Lanciego, Floris G. Wouterlood

**Affiliations:** 1 Neurosciences Department, Center for Applied Medical Research (CIMA), University of Navarra, Pio XII Avenue 55, 31008 Pamplona, Spain; 2 Centro de Investigación Biomédica en Red de Enfermedades Neurodegenerativas (CiberNed), Pamplona, Spain; 3 Instituto de Investigación Sanitaria de Navarra (IdiSNA), Pamplona, Spain; 4 Department of Anatomy and Neurosciences, Amsterdam University Medical Centers, Location VUmc, Neuroscience Campus Amsterdam, P.O. Box 7057, 1007 MB Amsterdam, The Netherlands

**Keywords:** *Phaseolus vulgaris*-leucoagglutinin, Biotinylated dextran amine, Fluorescent tracers, Cholera toxin, Viral vectors

## Abstract

Neuroanatomical tracing methods remain fundamental for elucidating the complexity of brain circuits. During the past decades, the technical arsenal at our disposal has been greatly enriched, with a steady supply of fresh arrivals. This paper provides a landscape view of classical and modern tools for tract-tracing purposes. Focus is placed on methods that have gone viral, i.e., became most widespread used and fully reliable. To keep an historical perspective, we start by reviewing one-dimensional, standalone transport-tracing tools; these including today’s two most favorite anterograde neuroanatomical tracers such as *Phaseolus vulgaris*-leucoagglutinin and biotinylated dextran amine. Next, emphasis is placed on several classical tools widely used for retrograde neuroanatomical tracing purposes, where Fluoro-Gold in our opinion represents the best example. Furthermore, it is worth noting that multi-dimensional paradigms can be designed by combining different tracers or by applying a given tracer together with detecting one or more neurochemical substances, as illustrated here with several examples. Finally, it is without any doubt that we are currently witnessing the unstoppable and spectacular rise of modern molecular-genetic techniques based on the use of modified viruses as delivery vehicles for genetic material, therefore, pushing the tract-tracing field forward into a new era. In summary, here, we aim to provide neuroscientists with the advice and background required when facing a choice on which neuroanatomical tracer—or combination thereof—might be best suited for addressing a given experimental design.

## Introduction: the legacy of the old school

If we go back in time 40 years, consider the state of technology in neuroanatomy in those days and compare with today, two distinctive developments become visible. The first is that 1980 marks the closure of an exciting first decade of neuroanatomical tracing with the revolutionary horseradish peroxidase (HRP) tracing technique. HRP tracing was introduced in 1971 and exploits centripetal neuronal transport in living neurons (Kristensson and Olsson 1971a, b). HRP tracing completely overtook the experimental tracing technique of the day that was based on making lesions followed by detection of the increased argyrophilia of degenerating axons through complex silver staining procedures (Nauta and Gygax 1951; Fink and Heimer 1967).

Around 1980, most axonal tracing occurred within a conceptual frame that was essentially one-dimensional. Neuroanatomy in those days was by tradition completely focused on the aspects of connectivity and cytoarchitectonics. One would simply study projections from area A to area B. Sections were Nissl-counterstained to reveal the underlying cytoarchitectonics, and that was it. Things started to change rapidly in the early 1980s after trailblazing work done by Van der Kooy and Steinbusch (1980). A body of reports began to accumulate in the literature describing two-dimensional tracing procedures in which tracing of connectivity was supplemented with immunohistochemical (functional) identification of neurons and brain areas (e.g., Van der Kooy and Sawchenko 1982; Fallon and Seroogy 1984; Grove et al. 1986; Staiger and Wouterlood 1990; Berendse et al. 1992a). Two- and even multi-dimensional tracing had been anticipated by Gerfen and Sawchenko (1985) when they published their innovative tracing method that utilized the uptake and transport of leucoagglutinating subunit complexes of the red kidney bean (*Phaseolus vulgaris*) lectin (PHA-L, Gerfen and Sawchenko 1984). PHA-L accounted for a true breakthrough, first because axon tracing with the lectin exploited the centrifugal component of metabolic neuronal transport systems, second because the lectin appeared being almost exclusively transported along the axons to the axon terminals (anterograde transport), and third because the detection of transported tracer was based on immunohistochemistry. As these three aspects gave PHA-L tracing superiority compared with existing methods, the lectin was subsequently exploited in a large number of one- and two-dimensional neuroanatomical studies. It should be mentioned at this point that immunohistochemical detection of transported PHA-L makes it relatively easy to combine tracing with a second immunostaining detection procedure, depending on the availability of proper antibody combinations. Thus, it can be stated that the introduction of PHA-L marked the beginning of the age of multi-dimensional anterograde tracing. Among the first to capitalize on the new opportunities were Grove et al. (1986) who, using PHA-L tracing combined with acetylcholinesterase staining, investigated striatal efferent projections with emphasis on juxtaposition of PHA-L labeled fibers and acetylcholinesterase positive (i.e., cholinergic) neurons in globus pallidus. The introduction of high-resolution confocal laser scanning microscopy (CLSM) imaging in the mid-1990s combined with the availability of antibodies conjugated to new, fading-resistant fluorochromes, made multi-dimensional tracing easier and more accurate than ever before (cf. Zaborszky et al. 2006). It can be argued that the bottom line in neuroanatomical tracing has evolved from one-dimensional studies in 1990 to at least two-dimensional work today.

The second aspect of axonal tracing anno 1980 was that it was embedded in a single, well-outlined discipline, i.e., neuroanatomy, with a few links with other disciplines. Also this type of one-dimensionality that was so typical for early- to mid-twentieth century science has been changed in a relatively compact time frame into multi-disciplinary approaches that integrate neuroscience, molecular, cell biology, genetic, and neurophysiological methods. Most progress in this respect has been experienced in tracing with viruses such as further outlined in the section “The slow, unstoppable rise of viruses as tracing tools”. The first of these agents (Herpes Simplex virus-1) had been introduced in the early 1970s as a one-dimensional, retrograde tracing tool (Kristensson et al. 1974). Viruses were further explored as neuroanatomical tracing tools by Ugolini and Kuypers (1986), Ugolini (1995), Strack and Loewy (1990) and, combined with genetics and molecular biology, took off after the turn of the twenty-first century in a spectacular way. Note that in the early studies, replication of the virus was the process driving the tracing method, with viral capsule antigen detection as the reporter. The introduction of adeno-associated viruses (AAV) as a delivery vehicle of altered genes to force neurons to express green florescent protein (GFP) (Peel et al. 1997) can be considered as one of the milestones in neuroscience, because it triggered transformation of the field from several essentially one-dimensional disciplines into the big, multi-dimensional molecular-genetic-tracing-functional spectrum that we experience today. A similar thing happened in parallel with neurophysiological and molecular-genetic methods which resulted in the current field of optogenetics.

## Neuroanatomical tracing with transport methods

**Fig. 1:**
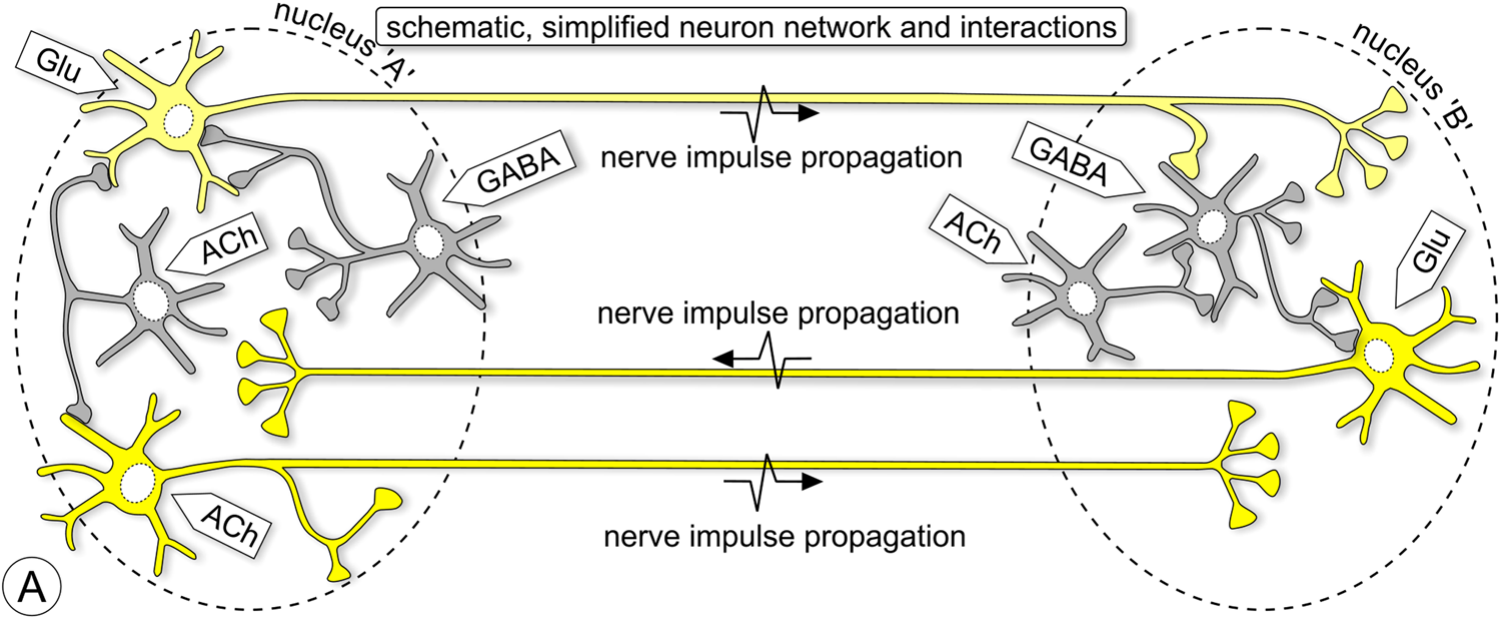
Scheme illustrating three issues addressed in this review: (1) axonal connectivity between brain nuclei, (2) neurochemical phenotyping; 3) microcircuits inside nuclei. This scheme is repeated as ‘template’ in Figs. 2, 5, 10, and 11 to illustrate which neurons can be expected to become labeled with various classical neuroanatomical tracers, viruses, toxins, and viral vector expressions. Nucleus ‘A’ has glutamatergic (Glu) and cholinergic (ACh) neurons (first-order) projecting to nucleus ‘B’, while the projection from ‘B’ to ‘A’ comes exclusively from glutamatergic (first-order) neurons. Nucleus ‘A’ contains (second-order) GABAergic and cholinergic interneurons; nucleus ‘B’ contains (second-order) GABAergic interneurons and third-order cholinergic neurons

**Fig. 2:**
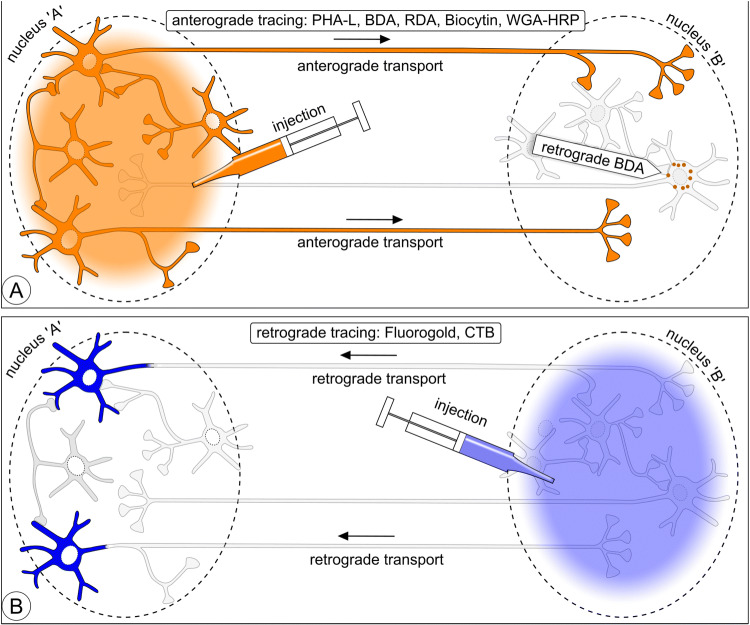
One-dimensional neuroanatomical tracing: **a** anterograde tracing. Tracers such as PHA-L, BDA, RDA, Biocytin, or WGA-HRP injected in nucleus ‘A’ label complete efferent fiber trajectories including axon terminals in nucleus ‘B’. Neurons in nucleus ‘A’ are labeled irrespective of their neurochemical phenotype. In the injection site projection, neurons and interneurons both become labeled and are indistinguishable from each other. WGA-HRP and BDA are to some degree retrogradely transported (indicated here as granules in the first-order neurons in nucleus ‘B’). **b** Retrograde tracing. Compounds such as Fluoro-Gold, CTB, RV, and PRV label projection neurons irrespective of their neurochemical phenotype

The aim of neuroanatomical tracing is to reveal the extremely complicated connectional relationships between neurons within the confinement of grey cerebral matter in a nucleus or cortical layer and, at a larger scale, connectivity between brain areas. The challenge is in its essence presented in Fig. 1: connectivity between two nuclei, each with its own set of projection neurons and interneurons. This scheme is used in Figs. 2, 5, 10, and 11 as a template, wherein the essentials of various tracing and marking/imaging techniques are explained.

**Fig. 3:**
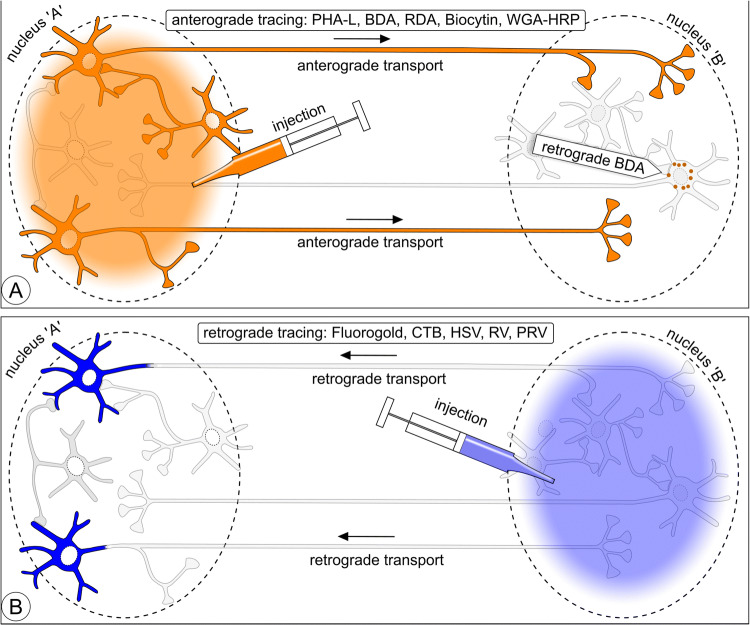
Anterograde tracing: injection sites and labeling. **a**, **b** Case 2012-6 (rat). In the same surgical session, we injected PHA-L in the central caudate putamen (CPu) of the left (L) cerebral hemisphere, while contralaterally (R), we injected BDA in the caudal CPu. Note significant retrograde transport of BDA-to-cerebral cortical pyramidal neurons ipsilateral to the injection site (dashed ellipse). Injection sites measure approximately 1 mm in diameter. **c** Low-power fluorescence montage in case FS-95166: injection site of PHA-L in the central nucleus (CE) of the amygdaloid complex. *Bmg* magnocellular basal amygdaloid nucleus. **d** Low-magnification montage of a combination of PHA-L tracing and neurobiotin injection. PHA-L-labeled fibers (red; Cy3) in layers II–III of medial parahippocampal cortex in contact with apical dendrites of neurobiotin-labeled pyramidal cells (green) located deep (layer V). *LD* lamina dissecans. **e** Low magnification: combination of PHA-L tracing and AF555 intracellular injection. An apical dendrite of an intracellularly AF555 injected hippocampal CA1 pyramidal neuron (red) penetrating stratum lacunosum moleculare (LM) The latter contains a terminal field of PHA-L labeled fibers (Alexa Fluor^®^ 488; green) belonging to the perforant pathway (PHA-L injected in medial parahippocampal cortex). *SR* stratum radiatum

**Fig. 4:**
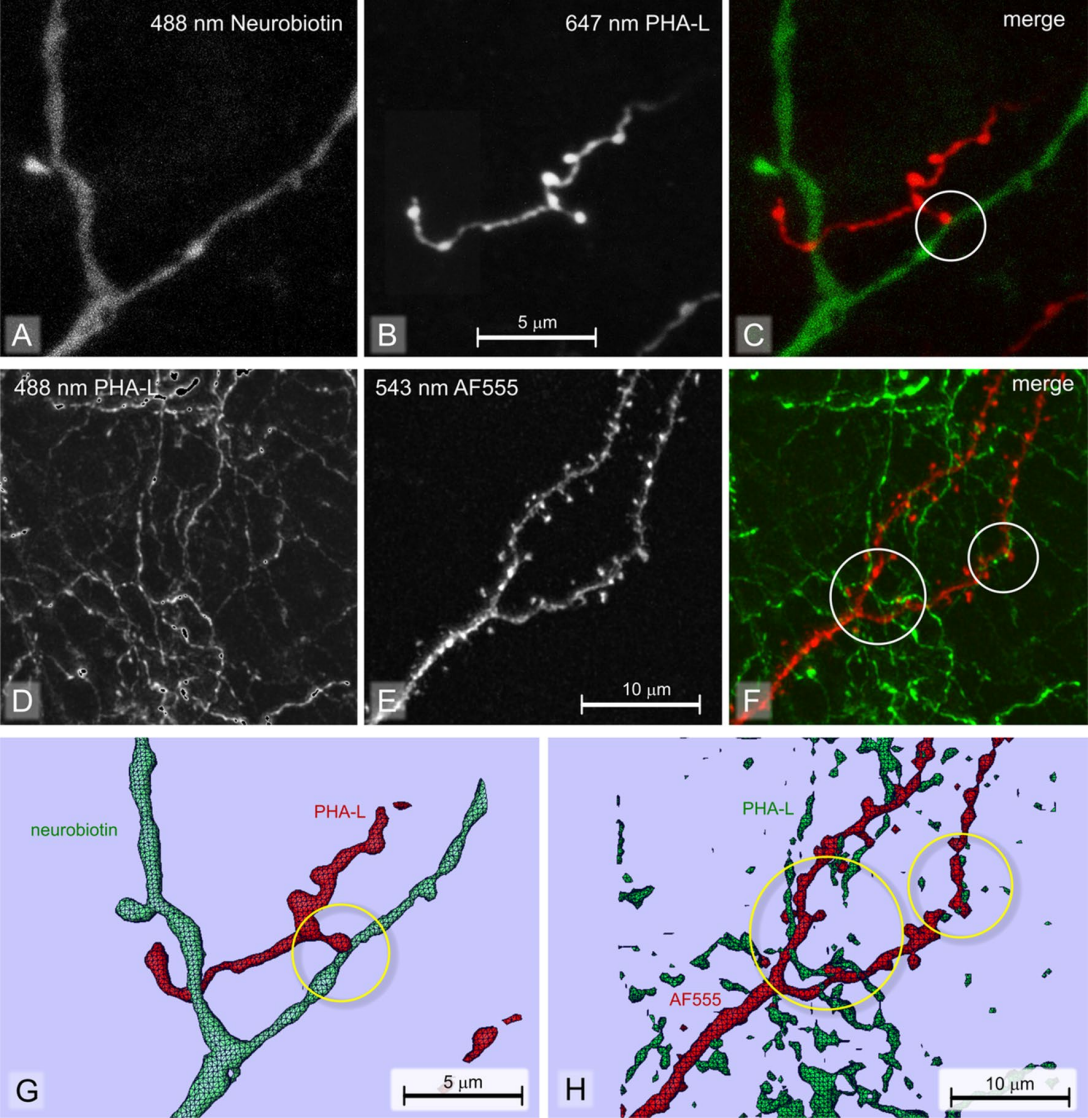
Two-dimensional neuroanatomical tracing. High-power confocal laser scanning images of the two-dimensional experiments illustrated in Fig. 3d, e. **a** Neurobiotin-labeled dendrite imaged in the green channel. **b** PHA-L labeled fiber (simultaneously) imaged in the infrared channel. **c** Merge. Encircled: an ending of a PHA-L labeled fiber (red) contacts a neurobiotin-labeled dendrite (green). **d** PHA-L-labeled perforant pathway fibers in stratum lacunosum moleculare imaged in the green channel, **e** AF555-labeled dendrite simultaneously imaged in the red channel. **f** Merge image; the circles indicate sites where boutons of PHA-L labeled fibers are in contact with the AF555 labeled dendrite. Images **a**–**f** are Z-projections. **g** 3D computer reconstruction made on basis of the images in (**a**, **b**). H, 3D computer reconstruction made on basis of the images in (**d**, **e**). Contacts are encircled

**Fig. 5:**
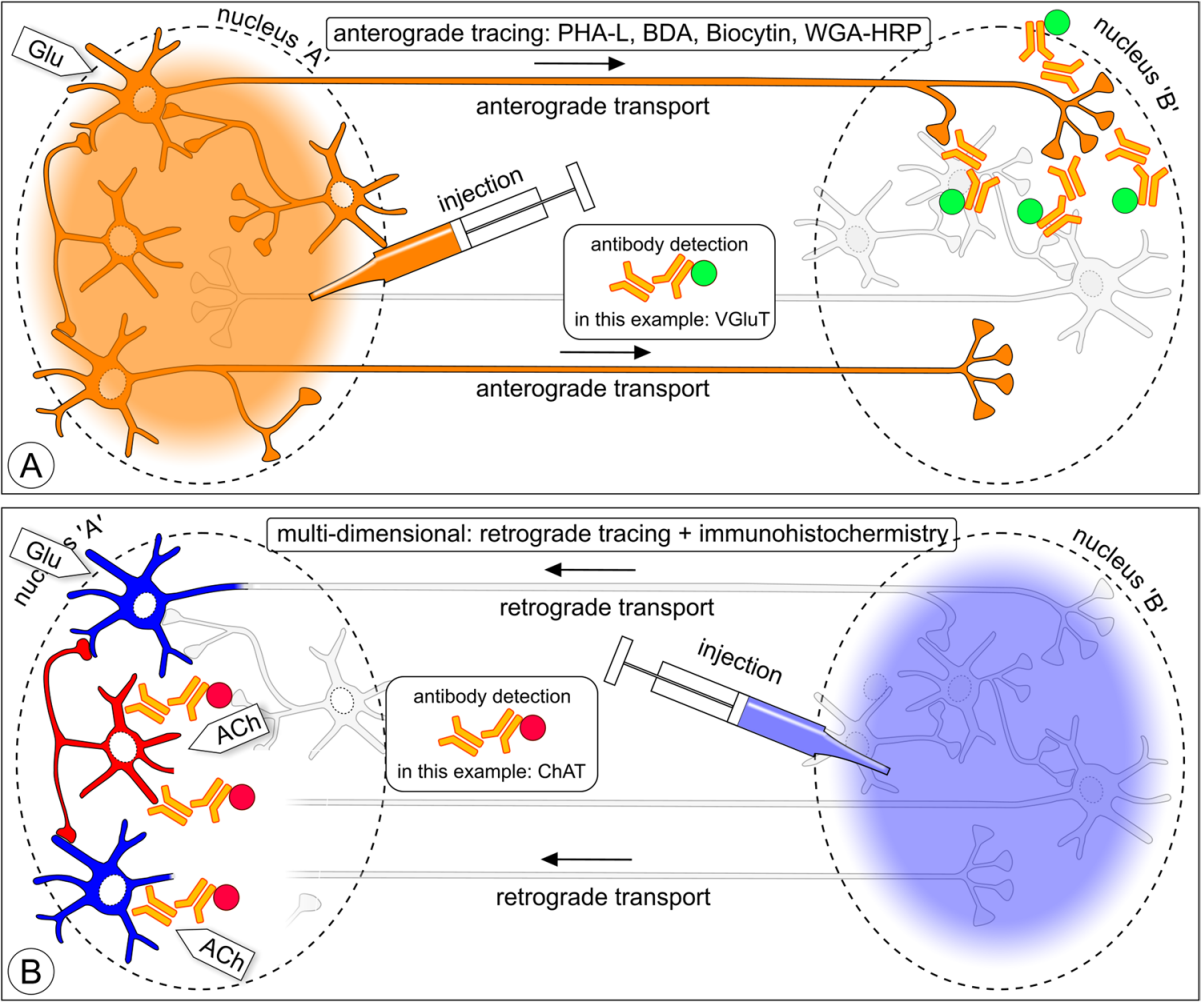
Multi-dimensional neuroanatomical tracing. A layer of immunohistochemistry is added to the tracing layer to distinguish neurochemical phenotypes. Many layers are possible. **a** ‘Neurochemical fingerprinting’: anterograde tracing with a suitable tracer followed by immunohistochemistry using an antibody to detect a marker inside tracer-labeled axon terminals. **b** Immunocytochemistry to reveal the neurochemical phenotype of retrogradely labeled neurons (in this example cholinergic neurons)

**Fig. 6:**
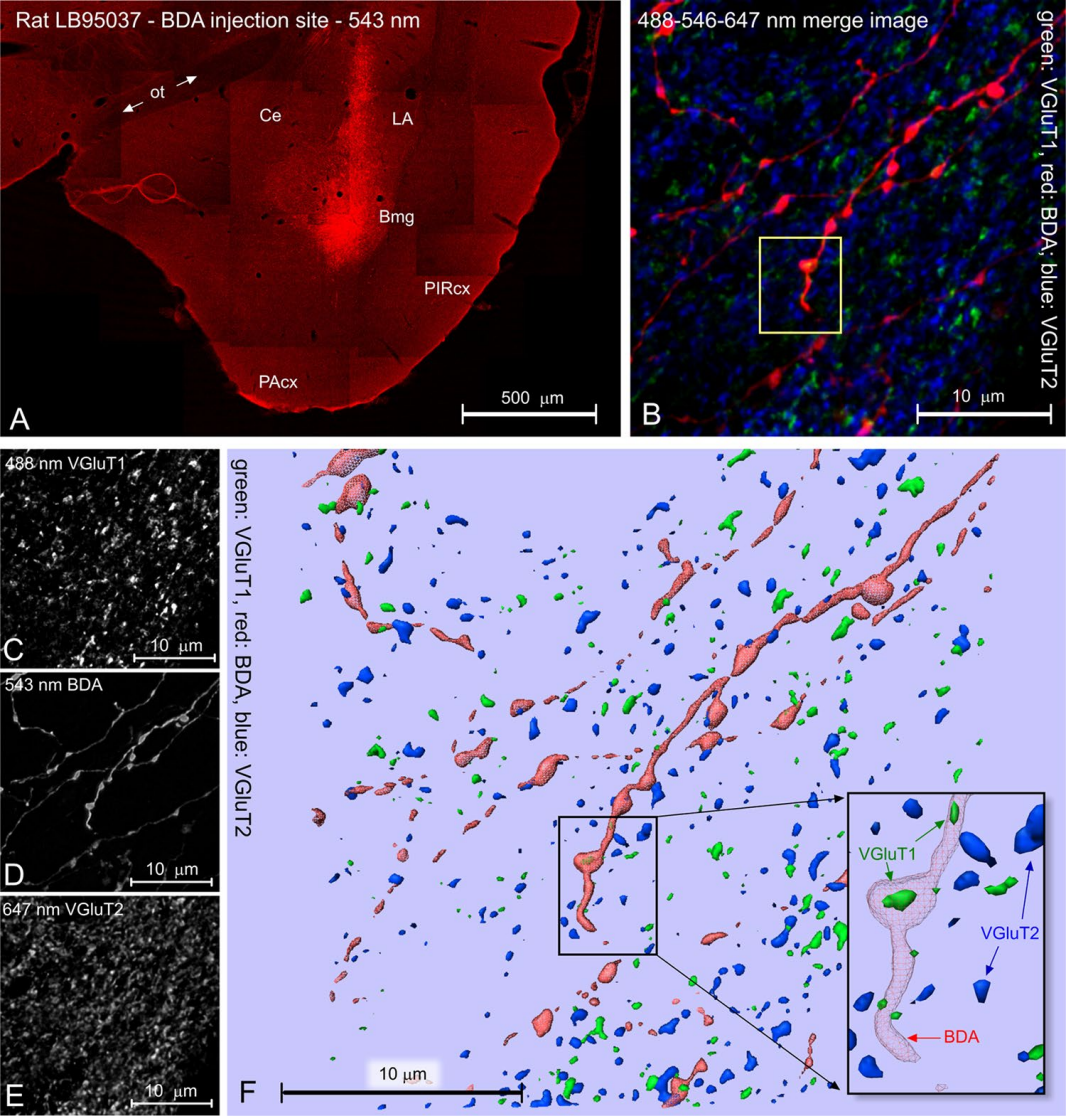
Multi-dimensional experiment. **a** Low-power image montage of the injection site of BDA in the basomedial nucleus of the amygdala (Bmg; rat LB95037). *Ce* central amygdaloid nucleus, *LA* lateral amygdaloid nucleus, *PAcx* periamygdaloid cortex, *PIRcx* piriform cortex, *ot* optic tract. **b** High-power imaging; sampling in striatum: Z-projection merge image of Z-stacks of images acquired at 488, 546, and 647 nm excitation in the confocal instrument. Color coding: green: VGluT1, red: BDA; blue: VGluT1. Boxed area: expression of VGluT1 inside a swelling (bouton) of a BDA-labeled fiber. **c**–**e** Z-projections of, respectively, the images acquired at 488 nm (VGluT1), 543 nm (BDA), and 647 nm (VGluT2). **f** 3D computer reconstruction. The inset is the fiber seen in the boxed area, frame (**b**). The BDA-labeled fiber (red) contains VGluT1 immunofluorescence (green) in both a bouton and in the fiber shaft (arrows). Notice that there is no colocalization of VGluT1 and VGluT2

**Fig. 7:**
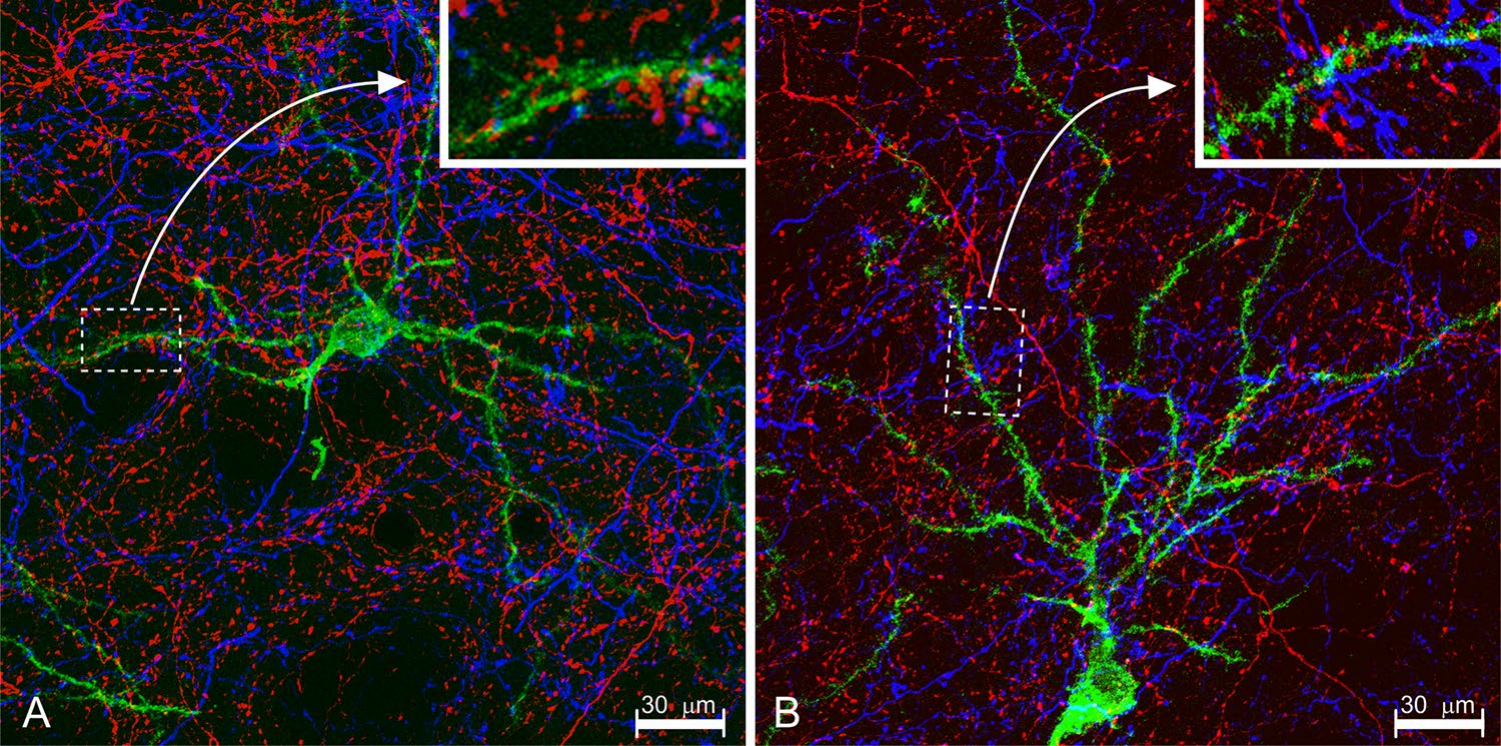
Multi-dimensional experiment combining in a rat dual-anterograde tracing with PHA-L (blue) and BDA (red) with retrograde tracing using rabies virus (RV; green). Here, we wanted to evaluate whether corticostriatal and thalamostriatal projections converge onto striatofugal neurons. PHA-L and BDA were iontophoretically delivered into the primary motor cortex and parafascicular thalamic nucleus, respectively. RV was pressure-delivered into either the external globus pallidus or in the substantia nigra pars compacta. **a** Striatopallidal; **B** striatonigral neuron. The insets show details. Detection of RV was carried out with an antibody against a soluble rabies phosphoprotein, resulting in Golgi-silver impregnation like labeling of dendrites, with details like dendritic spines

**Fig. 8:**
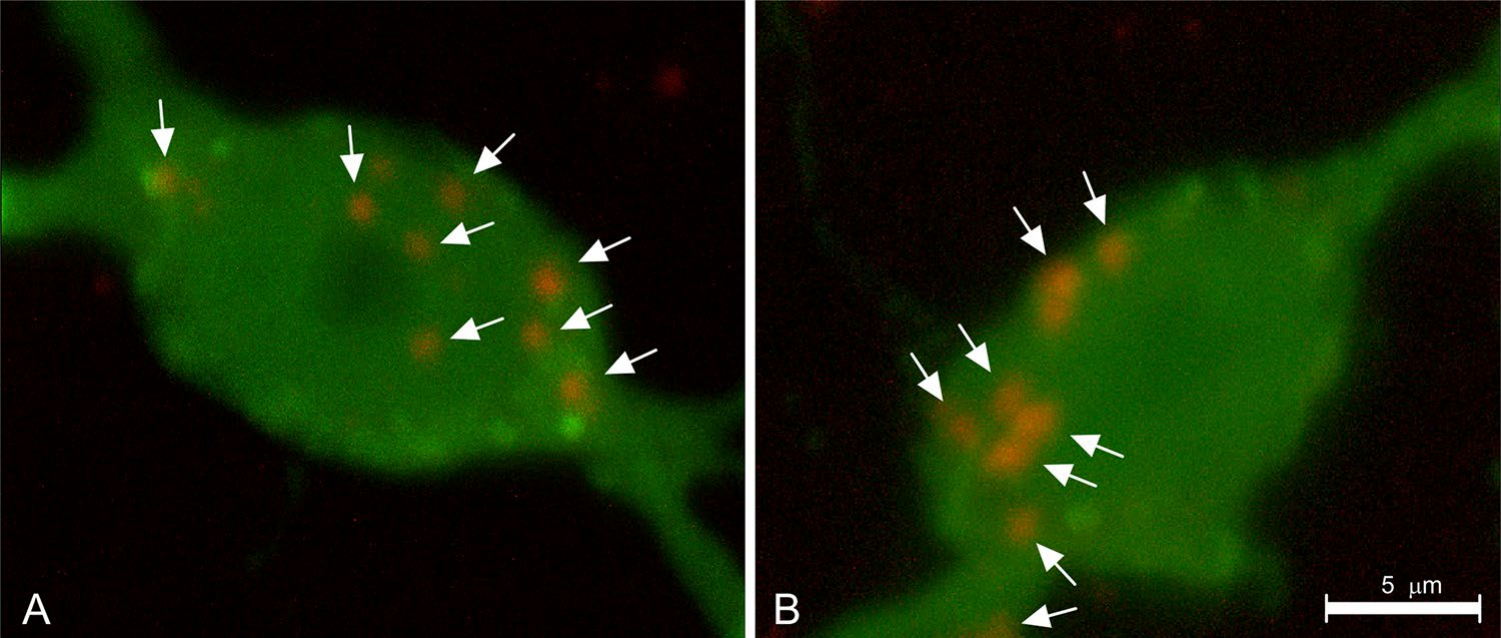
Multi-dimensional experiment combining retrograde BDA tracing (green fluorescence) and in situ proximity ligation assay (PLA, red fluorescence) in the non-human primate brain. Here, the goal was to identify the precise location of GPCR heteromers within striatopallidal projecting neurons. For these purposes, BDA was pressure-injected into the internal division of the globus pallidus, which was further visualized with an Alexa Fluor^®^ 488-tagged streptavidin. Next, the PLA technique was carried out for disclosing the precise location of GPCR heteromers within identified projection neurons within the post-commissural putamen. Each red dot represents one GPCR heteromer

**Fig. 9:**
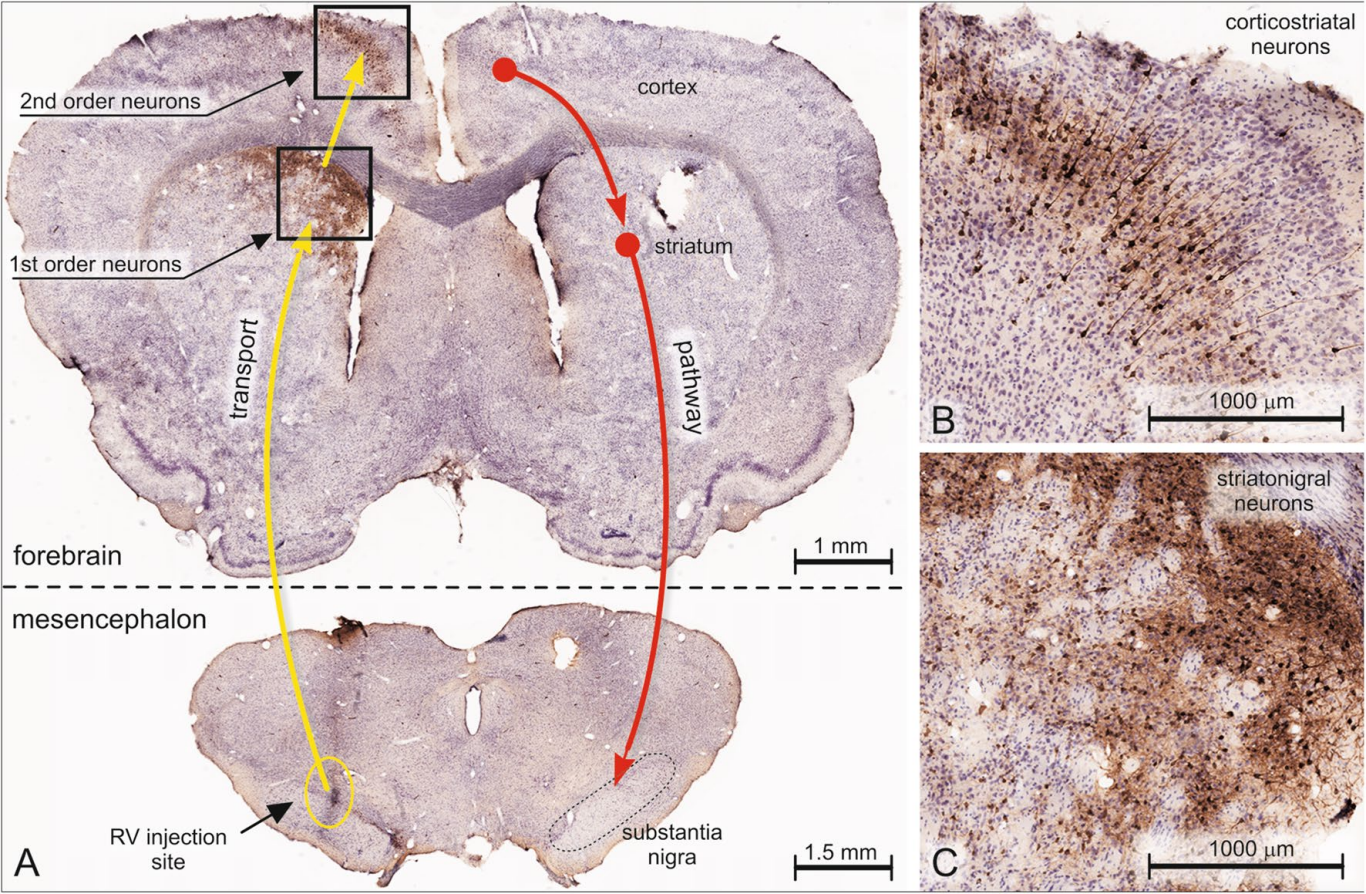
Transsynaptic retrograde tracing in rat with rabies virus (RV). **a** The investigated two-neuron chain schematically indicated on the right (red arrows): cerebral cortex to striatum, striatum-to-substantia nigra. The yellow arrows on the left indicate the retrograde tracing route. RV was pressure-injected into the substantia nigra. One week later, both first-order neurons (medium-sized striatal spiny striatonigral-projecting neurons) and second-order neurons (corticostriatal-projecting pyramidal neurons) could be observed. **b**, **c** Enlarged portions (the boxed areas in a). Retrograde transsynaptic spread of RV enables the accurate detection of neurons linking successively cerebral cortex, striatum, and substantia nigra. Detection of RV was with a primary antibody against a soluble rabies viral phosphoprotein, followed by a biotinylated IgG, then incubated with an HRP-tagged streptavidin and finally visualized with DAB

**Fig. 10:**
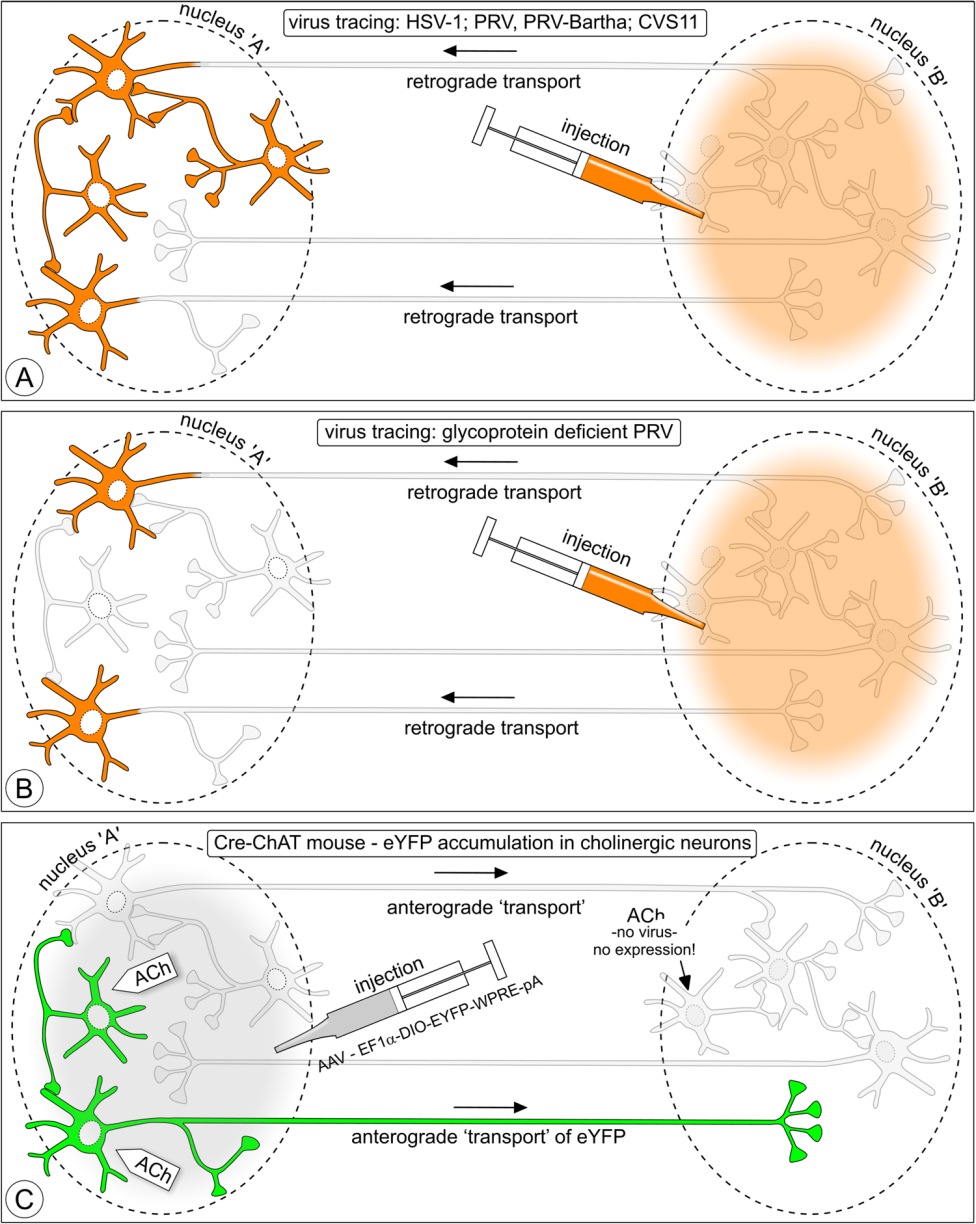
Advanced microcircuit visualization via a helper virus injection-rabies virus tracing paradigm according to Wickersham et al. (2007b). Schematic illustration of the components in the connectivity template of Fig. 1 that can be expected to become labeled. First-order neurons accumulate GFP (green), while the second-order neurons accumulate mCherry (red)

**Fig. 11:**
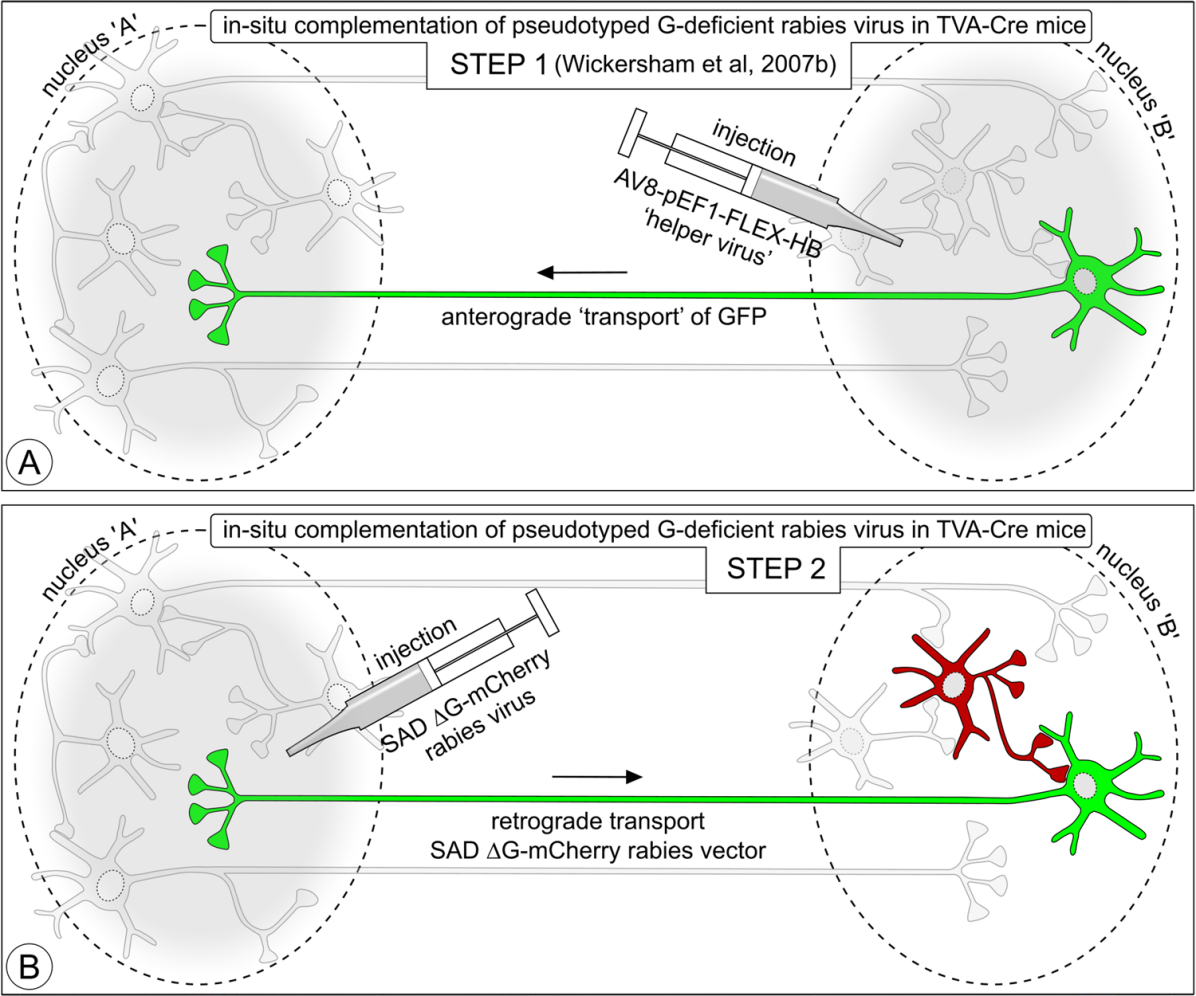
Components in the connectivity template of Fig. 1 that can be expected to become labeled when various neurotropic virus tracers and viral vectors are applied. **a** Classical neurotropic viruses: HSV-1, rabies virus, and swine rabies virus (PRV), retrogradely infect first-order neurons and proceed transsynaptically to second-order neurons, and so on. After several transfers, all neurons are infected and will stain. **b** Glycoprotein deficient virus lacks the gene that is responsible for transsynaptic transfer and, therefore, retrogradely labels only first-order neurons. **c** A focally injected, genetically engineered, non-replicating AAV virus infects a small population of neurons in ACh-Cre-dependent mice and inserts a reversed eYFP-coding gene in the genome of these neurons. Only neurons expressing a specific gene [in this case, the gene that codes for the choline acetyltransferase enzyme (ChAT) that synthesizes acetylcholine (ACh)] also express DNA recombinase that subsequently is responsible for ‘restoring’ eYFP expression. eYFP accumulates in all neuronal processes of these cells and acts as ‘anterogradely transported’ tracer. Cells that do not express the ChAT gene do not express DNA recombinase and, therefore, cannot express eYFP

### Anterograde axonal tracing with *Phaseolus vulgaris*-leucoagglutinin (PHA-L)

Four leucocyte agglutinating lectin subunits extracted from the red kidney bean (*Phaseolus vulgaris*) form a complex (PHA-L) that, deposited into a selected spot in grey matter in the CNS (for instance a cortical layer, subcortical nucleus, or spinal cord funiculus), is taken up by neurons. Label is homogeneously distributed inside the cytoplasm of the cell bodies, dendrites, and axons of labeled neurons. The large lectin molecules apparently link to the neuron’s internal transport system and get carried inside the axons all the way to the axon terminals (Fig. 2a). Detection is with antibodies against PHA-L.

The characteristics of PHA-L as an exclusive anterograde neuroanatomical tracer were described first by Gerfen and Sawchenko (1984). These authors noted that PHA-L fills neurons into all their nooks and crannies, revealing their most delicate morphological details (reviewed in Groenewegen and Wouterlood 1990). Detection is with a proper antibody. The reward of all the surgical and immunohistochemical effort is an exquisite, eye pleasing visualization of cell bodies, dendrites, and the entire trajectories of projection fibers including axon collaterals, terminal branches, and terminal boutons. PHA-L immunoreaction product viewed in the electron microscope is a homogeneously distributed electron dense substance occupying the matrix of the labeled fibers and axon terminals (Wouterlood and Groenewegen 1985; Smith 1992) [although this homogeneity depends to some degree on antibody penetration into the tissue sections, see Wouterlood et al. (1990)]. An attractive property of PHA-L is that, given suitable combinations of antibodies and chromogens in the immunohistochemical detection, multilabel experiments are within reach that may extract functional information from the labeled neurons. The prospect for such multi-dimensional experiments, wherein anterograde axonal tracing based on PHA-L transport would be one (principal) component, was already foresighted by Gerfen and Sawchenko in their second paper on the application of PHA-L (Gerfen and Sawchenko, 1985). It should be noted here that PHA-L does not naturally occur in brain tissue and for that matter does not contribute to background (if there is background staining, this is most probably caused by non-specific antibody binding). This ‘exoneuronal’ feature of PHA-L makes the tracer so well suited for combination with immunostaining of all kinds of neuroactive substances, metabolic enzymes, receptors, and transport proteins.

#### PHA-L tracing protocol

##### General

Animals are anesthetized and introduced into a stereotaxic apparatus. An opening is made in the skull, the meninges cut, and the surface of the brain exposed.

##### Injection

A glass micropipette with a tip opening of 10–30 μm in diameter containing 2.5% PHA-L in 50-mM phosphate buffer, pH 7.4, is lowered into the brain to the desired stereotaxic coordinates. If the tip-opening diameter exceeds 30 μm, a fair chance exists that an injection blob develops without signs of uptake and transport (Groenewegen and Wouterlood 1990). Very small injection spots including, say, 25 neurons are associated with the use of pipettes with tip diameter of 10 μm or less. Injection sites may measure up to 1000 μm in diameter (e.g., Fig. 3a), but their geometry depends on structural factors, e.g., the presence of bundles or sheets of myelinated fibers or the orientation of dendrites of local neurons. Application is iontophoretic, with a positive DC current of 10 μA, pulsed, 7 s on, 7 s off. The power source should be of the constant current type and capable of producing a high voltage to maintain the set current. After injection, the pipette is left in place for 10 min and then slowly retracted to avoid pipette track labeling.

That the uptake results in spread of label in particular in the cytosolic compartment of neurons suggests uptake via an electroporation phenomenon: the high-voltage iontophoretic pulses structurally change outer envelope membrane properties of neurons temporarily, that is, within a certain radius from the pipette tip position. The nanopores thus formed in the fluid membranes enveloping neurons allow externally deposited material to enter the cytoplasm. Electroporation is often used as a tool in many molecular biology transfection applications (Washbourne and McAllister 2002; Karra and Dahm 2010). The limited diameter of injection spots is consistent with the electroporation explanation. Once in the cytosol, the lectin must be picked up by intracellular transport systems, since one can define a ‘transport’ time.

##### Post-injection

Post-surgery survival time is not very critical. In rats, 1–3 weeks are usually sufficient to label intracerebral pathways. Transport speed is in the range of 5–6 mm per day (Gerfen and Sawchenko 1984). In rats, transported PHA-L can be detected with high quality up to 4 weeks after application, after which the details of labeling slowly start to deteriorate (Wouterlood et al. 1990). We routinely use for intracerebral connectivity experiments in rats a post-surgery transport time of 1 week. Next, the experimental animals are deeply anesthetized and transcardially perfused with 4% freshly depolymerized paraformaldehyde in 125-mM phosphate buffer, pH 7.6. A trace of glutaraldehyde (0.1–0.5%) may be added. We prefer a flush with carbon dioxide-bubbled Ringer solution at the beginning of the perfusion (cf. Friedrich and Mugnaini 1981). The brain is recovered from the skull immediately after the fixation procedure and either sectioned the same day on a vibrating microtome or equilibrated overnight in 20% DMSO-2% glycerin in phosphate buffer, pH 7.4, before cutting sections on a freezing microtome. Section thickness usually is 40 μm. Sections can be stored long-term at − 20 °C or colder in a cryoprotectant consisting of 20% glycerin and 2% dimethylsulfoxide in 100-mM phosphate buffer, pH 7.4 (Rosene et al. 1986). We have successfully stained sections of rat brain stored for more than 20 years under these conditions (Wouterlood et al. 2018a).

##### Incubation

Immunohistochemistry is routinely conducted with free-floating sections. The main incubation vehicle is TBS-TX: Tris-buffered saline, pH 8.0, with 0.5% Triton X-100 added. During all incubations, rinses, and reactions, the vials with the sections are gently rocked on a rocking plateau. Rinse between each step 3 × 10 min with TBS-TX unless otherwise specified.

##### Recipe for one-dimensional PHA-L detection

- Block 1 h in 10% normal rabbit serum in TBS-TX to suppress non-specific background.
- Incubate overnight in primary antibody (e.g., goat-anti-PHA-L,1:2000) at room temperature (or over the weekend in a refrigerator).
- Incubate 2 h in secondary antibody (here a 1:200 rabbit–anti-goat IgG).
- Incubate 1 h in a goat-peroxidase–antiperoxidase complex (1:800).
- Rinse 3 × 10 min in TBS-TX and twice briefly in TBS, pH 7.6 to remove the detergent.
- Transfer to 50-mM Tris, pH 7.6.
- Incubate in diaminobenzidine (DAB). DAB solution is prepared fresh by dissolving 5-mg 3-3′ diaminobenzidine-HCl (Sigma) in 10-ml 50-mM Tris–HCl, pH 7.6. After filtering, 3.3-μl 30% H_2_O_2_ (Merck) is added just before use. Hydrogen peroxide acts as the electron acceptor that powers the reaction (Graham and Karnovsky, 1965). Caution! Diaminobenzidine is a suspect carcinogenic.

After the immunostaining, the sections are mounted on gelatinized slides, dried, counterstained if necessary, and coverslipped with a synthetic mounting medium. Figure 3a shows a PHA-L injection site with the typical brown classical peroxidase staining reaction product, photographed at low magnification in a standard photomicroscope.

##### Immunofluorescence detection

Fairly soon after robust specific fluorochromes and powerful fluorescence imaging systems became available in the second half of the 1990s, we switched from classical immunoperoxidase staining to immunofluorescence staining. The latter involves standard incubation with the primary antibody: a goat or rabbit antibody against PHA-L. The next incubation step is a 2-h incubation in rabbit–anti-goat IgG (or goat–anti-rabbit IgG) conjugated to a fluorochrome with an excitation wavelength of choice. A typical PHA-L injection site in fluorescence imaging is shown in Fig. 3c. This particular image is a mosaic of six overlapping image ‘tiles’ acquired in a confocal laser scanning microscope. Each ‘tile’ of the mosaic is a Z-projection of ten confocal images taken at incremental equidistant steps along the optical axis of the confocal instrument. Today, the arsenal of commercially available secondary antibody-fluorochrome conjugates is immense; after initially applying carbocyanine fluorochromes (Cy2^®^, Cy3^®^, Cy5^®^), we started working with the Alexa Fluor^®^ series of antibody conjugates from which we select a particular member depending on the spectral characteristics of our confocal laser scanning instrument, e.g., an Alexa Fluor^®^ 488 conjugated secondary antibody that emits green fluorescence in a confocal laser scanning microscope when excited with monochromatic 488 nm laser light (e.g., Figure 3c).

#### Advantages and disadvantages

The main advantage of PHA-L is the sublime quality of detail that the labeling produces (Figs. 3c–e, 4b, d, 7). Transport is nearly exclusively anterograde. Retrograde transport manifests itself as granular deposits in neuronal cell bodies with conspicuous absence of cellular detail. There appears little if no uptake by fibers of passage (Gerfen and Sawchenko 1984). The tracer has been used in many mammalian species: rat, mouse (Cipolloni et al. 1985; Hoogland et al. 1991; Ma et al. 2016), hamster (Rhoades et al. 1989), guinea pig (Thompson and Thompson 1987), cat (Otake et al. 1988), squirrel monkey (Smith et al. 1990), and macaque monkey (Rockland 1995; Amaral et al. 2014; Morecraft et al. 2017), but also in amphibians (frog: Kulik et al. 1994), reptiles (Russchen and Jonker 1988) and birds (Husband and Shimizu 1999). Neuroanatomical tracing with PHA-L in fish species is conspicuously absent in the literature.

One handicap in using PHA-L is that iontophoretic application is necessary through a micropipette. Injection spots are, therefore, small. Single, pressure injections of larger volumes have been reported; however, these often produce a central necrotic spot at the deposition locus. A workaround if larger areas need to be supplied with tracer is to cover such an area with multiple small injections.

If iontophoretic application fails for some reason, one may experience after staining a disappointing ‘cloud’ of brown reaction product in the injection spot instead of labeled neurons and neuronal processes. A suspected cause here is an irregular micropipette tip. Taken together, in all our PHA-L tracing experiments over the years together, we experienced a global deposition failure rate of, say, 10%.

In non-human primates, in contrast to its efficacy in rodents (e.g., corticospinal tract tracing in rat; Liang et al. 1991), regardless of transport distances, anterograde labeling with PHA-L seems primarily valuable to study short-terminal projections (Morecraft et al. 2009, 2017). An impressive study with intracerebral fiber connectivity in primates is Amaral et al.’s (2014) work in newborn macaque monkeys, wherein three tracers were tested: a radioautographic tracer (discussed in Section“Tritiated amino acids and radioautography”), PHA-L, and BDA [the latter discussed in detail in section “Axonal tracing with biotinylated dextran amine (BDA)]”.

One may consider the immunohistochemical detection of transported PHA-L as a disadvantage when a multi-dimensional approach is planned, since one may run out of suitable combinations of primary and secondary antibodies, especially when the detection of a second marker or third marker requires a highly specific or exclusive primary antibody.

#### Multi-dimensional tracing with PHA-L

Multi-dimensional tracing includes neuroanatomical tracing combined with one or more additional techniques designed to specifically visualize a second marker or third marker. Such a marker can be one that defines cytoarchitectonic borders, an additional anterograde or retrograde tracer, a neurotransmission-related marker, or some marker that identifies potential post-synaptic neurons and their processes. Originally, research of this type was conducted with non-fluorescent reporter markers (Wouterlood et al. 1987; Antal et al. 1990; Lanciego et al. 2000; Lanciego and Wouterlood 2011), while Aarnisalo and Panula pioneered with fluorescence reporting (Aarnisalo and Panula 1995). An example that suits the modern ‘fluorescence age’ combines PHA-L tracing with labeling of a small population of prospective target neurons with neurobiotin (Figs. 3d, 4a–c, g). Neurobiotin as a tracer is discussed in section "Biotin derivatives and carbocyanine dyes".

##### Labeling of axon terminals and their (presumed) post-synaptic targets

Once a bundle of fibers is labeled with PHA-L and the distribution of individual fibers in the target areas of the fiber projection is charted, one may want to identify neurons post-synaptic to the labeled axon terminals. For this purpose, PHA-L tracing can be combined with an additional technique that produces specific label inside neurons that may be targeted by the PHA-L-labeled projection. Often applied is a double-immunohistochemical procedure that includes detection of PHA-L and detection of the secondary marker. The latter is usually done via an antibody that highlights a specific type of neuron (presumed GABAergic, peptidergic, cholinergic, or dopaminergic targeted neurons, to name just a few). Initial attempts in this realm included experiments with ‘absorption’ tags, that is visualization with different light absorption (‘colored’) chromogens such as diaminobenzidine (brown precipitate), nickel-enhanced diaminobenzidine (black precipitate), tetramethylbenzidine (dark blue precipitate), and Vector VIP™ (magenta precipitate) (Wouterlood et al. 1987; Lanciego et al. 1997, 2000; Köbbert et al. 2000; Gonzalo et al. 2001; Lanciego and Wouterlood 2011 (review); Unal et al. 2014), while the introduction of confocal laser microscopy, and in parallel the introduction of robust fluorochromes, delivered resolution high enough to reliably detect markers even inside thin fragile fibers and axon varicosities (Wouterlood et al. 2007). Most work, nowadays, published in this field is documented with confocal microscopy (e.g., Boulland et al. 2009; Barbier et al. 2018). While a sequential procedure for detection of the involved markers is possible (e.g., Barbier et al. 2018), we prefer incubations with antibody cocktails to economize on the time running an experiment. We briefly present two examples.

*Example A* is from a study in the rat of fiber projections from the presubiculum to medial parahippocampal cortex. The presubiculum mediates transfer of information from retrospenial cortex to the hippocampus (Kononenko and Witter, 2012). The recipients here are the apical dendrites in superficial layers of parahippocampal neurons whose cell bodies are located deep in layer V (reported in Wouterlood et al. 2004).

*Example B* deals with appositions of perforant pathway terminal fibers and axon terminals with dendritic branches that are part of apical tufts of hippocampal CA1 pyramidal neurons. The perforant pathway was labeled in rats via injection of PHA-L in layers II–III of medial parahippocampal cortex, while the hippocampal CA1 neurons were intracellularly injected with Alexa Fluor^®^ 555 (AF555) (Molecular Probes, Eugene, OR). The entire procedure and the obtained results were published by Kajiwara et al. (2008).

##### Protocol

Injections with PHA-L were made in their respective loci according to the procedure outlined in the section ‘Injection’ of the PHA-L tracing protocol. The rats of example A were 1–4 days later, but always 48 h prior to sacrifice, re-anesthetized and re-introduced in the stereotaxic frame. A micropipette with a tip-opening diameter of 5–10 μm was filled with Neurobiotin™ and lowered to the desired position in the deep layers of the ipsilateral medial entorhinal cortex (MEA). Neurobiotin and PHA-L were both iontophoretically injected, with identical parameters (see PHA-L tracing protocol).

In both examples, we cut the brains, after sacrifice according to the PHA-L tracing procedure described above, into sections on a vibrating microtome. Standard section thickness is 40 μm, but in the experiments in which we applied intracellular injections (example B), we cut slices of 400-μm thick alternated with 100 μm-thick slices. During all incubations, rinses, and reactions, the vials with the sections are gently rocked on a rocking plateau. Rinse between each step 3 × 10 min with TBS-TX unless otherwise specified.

*Example A*: (double staining of free-floating sections)

- Block 1 h in 5% normal goat serum in TBS-TX.
- Incubate 48 h with rabbit anti-PHA-L antibodies (1:2000 in TBS-TX in a refrigerator).
- Incubate 1 h with a cocktail of 1:400 goat-anti-rabbit-Cy5^®^ mixed with streptavidin-Alexa Fluor^®^ 488 (in TBS-TX).
- Rinse the sections 3 × 10 min in TBS-TX, then briefly twice in TBS and then transfer to Tris–HCl, pH 7.4.
- Park in Tris–HCl, pH 7.4 for a short period awaiting mounting and coverslipping.

*Example B:*

- Place slices containing hippocampus in a Petri dish in a fixed-stage microscope equipped with a three-axis micromanipulator (Buhl and Lübke 1989).
- Approach cell bodies of CA1 neurons and impale them: glass micropipettes (tip-opening diameter was 0.5–0.8 μm) attached to a micromanipulator. Micropipettes contain 10-mM Alexa Fluor^®^ 555 hydrazide (AF555) in phosphate buffer, pH 7.6. Inject via microiontophoresis (2-nA current). Successfully impaled/injected CA1 neurons light up red under green fluorescence illumination. AF555, once introduced in a neuron, acts as a robust intrinsic fluorescent staining that does not need additional enhancement and that survives the subsequent immunofluorescence incubation procedure,
- Resection the slices after injection into a series of 50 μm-thick second-generation sections on a freezing microtome.
- Incubate 72 h in 1:2000 anti-PHA-L raised in rabbit, in TBS-TX.
- Incubate 2 h in 1:400 goat–anti-rabbit-Alexa Fluor^®^ 488, in TBS-TX.
- Rinse the sections 3 × 10 min in TBS-TX.
- Rinse briefly twice in TBS to remove the detergent.
- Rinse in Tris–HCl, pH 7.4, and park in this medium for a short period awaiting mounting and coverslipping.

##### Mounting and coverslipping procedure for fluorescence imaging

Sections were recovered from the parking, once more rinsed in Tris–HCl, mounted on 1% gelatin (Oxoid) coated glass slides and then thoroughly dried. This drying is critical for confocal microscopy, since traces of water will render the sections opaque. Imperfectly cleared sections will hamper imaging efficiency, because once a laser excites the fluorochrome molecules, a sizable fraction of the emitted photons will become absorbed by the tissue and, hence, never reach the detector.

Coverslipping is done by first dipping the slides with the thoroughly dried sections in analytical grade toluene for 30 s. Immediately afterwards, a drop of synthetic resin is applied and the coverslip put in place. The sections are then ready for immediate inspection in a fluorescence microscope. However, to avoid contamination of the microscope with sticky resin, it is advised to dry the slides first for 24 h in a dark drying cabinet at 30–40 °C. Final storage is in a freezer at − 20 °C.

##### Imaging

Slides belonging to examples A and B were studied in a confocal laser scanning microscope (CLSM; Leica TCS-SP2 AOBS). This instrument is equipped with 488-, 546-, and 647-nm lasers. 488-nm laser light was used to excite Alexa Fluor^®^ 488 associated with neurobiotin (example A) or PHA-L (example B); 647-nm laser light excited the Cy5 attached to the PHA-L labeled fibers (example A), whereas the 546-nm laser light excited AF555 in the CA1 neurons of example B. The instrument was always operated in sequential Z-scanning mode in two channels (example A: ‘green’ channel; excitation 488 nm, emission peak at 519 nm and ‘infrared’ channel: excitation 647 nm, emission peak at 670 nm; example B: ‘green’ channel; excitation 488 nm, emission peak at 519 nm and ‘red’ channel: excitation 546 nm, emission peak at 568 nm). The objective lens was a 63× plan apochromat NA 1.3 glycerin immersion lens; electronic zoom was 8×; and image dimensions were 512 × 512 pixels, 256 grey intensity levels (8 bit). Y-stepping was a calibrated 55 nm. Structures were 3D rendered using Amira™ software (Thermo Fisher Scientific). Results are illustrated in the low-magnification photomontage of Fig. 3d–e, and in the high-magnification images of Fig. 4a–f.

##### Note on diffraction-limited imaging

Study of contacts between tracer-labeled axon terminals (diameter 0.5–0.8 μm and prospective post-synaptic target neurons (dendritic spine size approximately 0.5 μm) requires high magnification. Here, we descend deeply into the blurry realm of Abbe diffraction limitation, wherein structures require super-resolution imaging to make them properly visible. To improve the resolution of our imaging system, we digitally processed the acquired image stacks with dedicated deconvolution software (Huygens™; SVI, Hilversum, The Netherlands) prior to 3D reconstruction (i.e., all high-magnification images shown in this paper).

#### Analysis of acquired images

Already at low magnification, the PHA-L-labeled fibers in sections in both examples formed a band of terminal fiber labeling. Presubiculum fibers ending in medial parahippocampal cortex (example A) occupy layers II–III densely, while layer I has scattered fibers (Fig. 3d). Perforant pathway labeling in hippocampal field CA1 (example B) consists of a very well-delineated dense ‘cloud’ in stratum lacunosum moleculare (LM, Fig. 3e). At high resolution, numerous occasions could be seen of swellings of PHA-L-labeled fibers (interpreted as boutons, axon terminals) in apposition (‘contact’) with dendrites labeled with the second label (Fig. 4c, f and the 3D reconstructions in Figs. 4g, h). Physical contacts of this type are necessary anatomical substrates in terms of pre- and post-synaptic structures. We demonstrated the presence of synapses separately, with electron microscopy (Wouterlood et al. 2004). Similar observations of appositions were made by Kononenko and Witter (2012) in their study of recipient fibers in layer III of medial parahippocampal cortex. The presence of a contact does not necessarily mean, however, that there is a functional synapse; additional verification is required. In a separate study (Wouterlood et al. 2003), we have shown the presence of the typical post-synaptic density anchoring protein, ProSAp1/Shank B (Boeckers et al. 1999; Raab et al. 2010) sandwiched in between labeled presynaptic fibers, and presumed post-synaptic neurons. Such a sandwich more convincingly indicates the presence of a synapse than ‘merely’ an apposition

### Axonal tracing with biotinylated dextran amine (BDA)

The early transport-tracing methods leaned on cellular centripetal transport mechanisms. The need to expand the repertoire of anterograde techniques beyond radioautographic methods (see the section “Tritiated amino acids and radioautography”) and (bidirectionally) transported WGA-HRP (see the section “Non-fluorescent and fluorescent retrograde tracing”) was satisfied in 1984 with the introduction of PHA-L (Gerfen and Sawchenko 1984, 1985). Research continued afterwards to find alternative tracers. After Glover et al. (1986) had introduced dextran amine conjugated to selected fluorescent dyes as a novel, yet retrograde, neuroanatomical tract-tracing macromolecule, dextran amine again came into the spotlight when a major anterograde transport component was reported (Nance and Burns 1990; Schmued et al. 1990; Fritzsch and Wilm 1990; Chang et al. 1990). Veenman et al. (1992) publication, immediately followed by one authored by Brandt and Apkarian (1992), brought biotinylated dextran amine (BDA) to center stage as an anterograde neuroanatomical tracer. Dextran molecules, dextran amines, and their conjugates are taken up via an unknown mechanism by dendrites and neuronal cell bodies, and transported to a large degree in the anterograde direction (Reiner et al. 2000; Reiner and Honig 2006) (Fig. 2, schematic). Biotinylated dextran amine conjugated to lysine, MW 10 kD (supplier Invitrogen-Molecular Probes, Eugene, OR) is the most popular among the dextran amines. BDA has been used as tracer in all classes of vertebrates, including fish species (Xue et al. 2001; Northcutt 2011).

#### BDA tracing protocol

##### Application

BDA is dissolved prior to use in 10-mM phosphate buffer, pH 7.25 (5–10%, rodents; 10%. primates). This solution is usually injected under stereotaxic guidance into the CNS via iontophoresis or through a mechanical injection (Reiner et al. 2000).

For small rodents, a glass micropipette (tip-opening diameter 20–40 μm) is filled with BDA solution. Typical iontophoretic application into rat brain is through a 5-μA positive pulsed direct current (7 s on/off) on. The same equipment as for PHA-L injections can be used.

Survival time is in proportion to the length of the projection under study. Transport is estimated to span 15–20 mm of tract in 1 week (Reiner et al. 1993). In rats, we use 1 week of survival time. BDA remains stable in the rodent brain up to 4 weeks post-injection, while in primates, it may remain detectable up to 7 weeks after application.

##### Fixation and detection

BDA tolerates a wide variety of fixatives. This is important if electron microscopy is on the horizon. Because BDA detection depends on penetration of tagged streptavidin into the tissue sections instead of (much larger) antibodies, a better compromise between penetration and preservation of ultrastructure can be obtained than with PHA-L (Wouterlood and Jorritsma-Byham 1993). In rodents, we prefer for light microscopy a fixative that allows additional immunohistochemistry: buffered 4% formaldehyde, 0.1% glutaraldehyde, and 0.25% of a saturated picric acid solution. After fixation, the brain can be cut with any of the available sectioning methods. We have processed sections with thickness ranging between 40 and 400 μm.

Detection of transported BDA is very simple and straightforward: incubation with one of the many commercially available tagged (strept)avidins. An irreversible reaction occurs between biotin and avidin that results in a stable product. The tag can be a fluorochrome, horseradish peroxidase, or even biotin (followed by incubation with biotinylated streptavidin to amplify signal). While fluorochrome-tagged, bound avidin can be seen directly in a fluorescence microscope, visualization of bound horseradish peroxidase needs an extra diaminobenzidine-peroxide chromogen reaction.

If application fails for some reason, the sections usually look similar to failed PHA-L application: a vague ‘cloud’ of brown reaction product or some disappointing diffuse fluorescence in the injection spot instead of discrete, labeled neurons, and neuronal processes. However, BDA in tracing experiments less frequently fails in our experience than PHA-L.

##### Recipe for one-dimensional BDA detection

The main incubation vehicle is TBS-TX (Tris-buffered saline, pH 8, 0, with 0.5% Triton X-100 added). During all incubations, rinses, and reactions, the vials with the sections are gently rocked on a rocking plateau. Rinse between each step 3 × 10 min with TBS-TX unless otherwise specified.

- Incubate with streptavidin-peroxidase 1:400: overnight at room temperature, or 24 h in a refrigerator.
- Rinse twice in TBS, pH 7.6, to remove the detergent.
- Rinse in 50-mM Tris, pH 7.6.
- Conduct a monitored incubation in diaminobenzidine (DAB). DAB solution is freshly prepared by dissolving 5 mg 3-3′ diaminobenzidine-HCl (Sigma) in 10-ml 50-mM Tris–HCl, pH 7.6. After filtering, 3.3-μl 30% H_2_O_2_ (Merck) is added just before use.
- Rinse and park in 50 mM Tris, pH 7.6.

Mounting and coverslipping are identical to the procedure with sections in PHA-L tracing.

#### Results with BDA tracing

In a neuron that has taken up BDA, the tracer fills the matrix homogeneously. Background staining usually is negligible (this is extremely helpful in high-resolution confocal laser scanning microscopy where every emitted photon that reaches the microscope’s detector counts). All the details of the labeled neurons in the injection sites, e.g., dendritic spines, are available for inspection. Most important is that for the eye, the tracer appears equally distributed along the entire trajectories of fibers. This feature allows extremely precise mapping of fiber tracts, the analysis of the compartmentation of large fascicles and association bundles, and the study of terminal projection patterns. In the electron microscope, the label generated by BDA processing occurs in cytoplasmic compartments of the perikaryon, the main dendrites, their branches, branchlets, and spines, and in fibers, varicosities, and axon terminals. Nuclei sometimes contain BDA-reaction product. When utmost care is taken, ultrastructural detail can be preserved so well that synaptic vesicles and pre- and post-synaptic membrane densities of labeled axon terminals can be appreciated (Wouterlood and Jorritsma-Byham 1993). Retrograde transport of BDA may occur, resulting in a granular deposit of the tracer in a limited number of neuronal perikarya but sometimes complete or ‘dense’ labeling is present of cell bodies and dendrites in areas known to project to the site of injection (Reiner et al. 1993). In particular, pyramidal neurons in cerebral cortex show a tendency to accumulate retrogradely transported BDA. For instance, in experiment 2012-08 shown in Fig. 3a, b, cortical pyramidal neurons became retrogradely labeled after injection of the tracer into the caudate putamen. When such retrograde labeling occurs, the investigator should be aware of the possibility of ‘false’ anterograde labeling of collateral fibers of these, initially retrogradely labeled, neurons (see also Chen and Aston-Jones 1998).

#### Advantages and disadvantages

BDA tracing has a high success rate and the detection of transported tracer does not require antibodies. In addition, the tracer is very tolerant to fixative composition. These three features render BDA highly efficient and make it attractive to apply BDA in multi-dimensional tracing studies, even in spite of the disadvantage of the retrograde transport component. The latter was even exploited by Bácskai et al. (2010) who applied two fluorescent dextran amine derivatives: tetramethylrhodamine dextran amine (RDA) and fluorescein-dextran amine (FDA), contralateral to each other to the cut ends of the hypoglossal nerve in the frog, *Rana esculenta*. The experiment was conducted to study the relationships of hypoglossal motoneurons across the midline of the brain stem.

Its versatility, together with the ease of application, the straightforwardness, and speed of the staining protocol, and on top of this the reliability, even compared with PHA-L, has made BDA a widely applied and successful neuroanatomical tracer. At present, BDA undoubtedly represents the first choice anterograde tracer (Lanciego and Wouterlood 2011). BDA also compares good with modern, recombinant virus-based tracing (Wang et al. 2014).

The availability of multiwavelength confocal laser scanning microscopes with good signal separation has made it possible to conduct, for instance, three-dimensional experiments, e.g., application of BDA and PHA-L as tracers plus an additional marker pinpointing a neurotransmitter or other neuroactive substance. Injection of BDA in locus A and, in the same surgical session, of PHA-L in locus B (Herrera et al. 1994) opens ways to study in detail the anatomical convergence or divergence of neuronal connectivity. It also allows the study of connectivity in long neuronal circuits (reviewed in Lanciego and Wouterlood 2006). In non-human primates, and in contrast to its efficacy in rodents, regardless of transport distances, anterograde labeling with PHA-L seems primarily valuable to study short-terminal projections (Morecraft et al. 2009).

#### Multi-dimensional BDA tracing: neurochemical fingerprinting

Once a bundle of fibers is labeled and the distribution of individual fibers in an area where fibers project to is made visible, it may be worth to determine whether a particular neurotransmission-related marker is present inside the labeled fibers and terminals. This type of functional identification is further called ‘neurochemical fingerprinting’. Noteworthy, neurochemical fingerprinting *avant la lettre* was conducted in 1995 by Aarnisalo and Panula on possible colocalization in PHA-L-labeled medial hypothalamic neuron fibers of neuropeptide FF. The researchers used available, classical FITC–TRITC double immunofluorescence histochemistry, while imaging was achieved with standard fluorescence microscopy. Two conditions have to be met, however, to convincingly conduct this type of research. The double immunohistochemistry should be done with robust fluorochromes, while second and most importantly, high-resolution confocal microscopy is required, because only this kind of microscopy is capable of imaging colocalization with high confidence in very small, diffraction-limited objects such as axon terminals. Both PHA-L and BDA can be used as the projection fiber marker. For instance, Gautier et al. (2017) combined tracing with PHA-L as axonal tracer with 5HT immunohistochemistry to study serotonergic bulbospinal projections in rats. We prefer BDA as the axonal tracer, because its visualization does not depend on immunohistochemistry, leaving us with maximum immunohistochemical maneuverability to detect the second or third marker, while we operate at the limits of light microscope resolution.

The direct streptavidin-based detection of transported BDA detection represents added value, because it greatly facilitates application of BDA in multi-dimensional studies. Experiments may include multiple tracing paradigms as well as several molecular biology protocols. An example of a multiple tracing paradigm is the combination of anterograde BDA tracing with dual-retrograde tracing with cholera toxin subunit B (CTB) and Fluoro-Gold (FG), both in rodents and in non-human primates (Lanciego et al. 1998a, b; Erro et al. 1999; Lanciego et al. 2000, 2004; Castle et al. 2005). Furthermore, BDA performs nicely when combined with PHA-L (double anterograde tract-tracing, see Lanciego and Wouterlood 1994), or with PHA-L as two anterograde tracers in combination with retrograde tracing with rabies virus (triple neuroanatomical tracing; Fig. 7a, b; see Salin et al. 2008; López et al. 2010). Finally, it is noteworthy that BDA can also be combined with newly available tools for molecular biology, such as the so-called in situ proximity ligation assay (PLA). Initially introduced by Söderberg et al. (2008), PLA was designed to disclose interactions between two proteins when located very close to each other, enabling the accurate localization of places where these interactions are taking place. In the past few years, PLA has become increasingly popular for the visualization of heterodimeric complexes made of two different G protein-coupled receptors (GPCRs). In this regard, the combination of PLA and retrograde tracing with BDA allows the identification of GPCR heteromers within neurons innervating a given brain area where BDA was delivered (example E; Fig. 8A,B; Rico et al. 2017).

*Example C: VGluT1 or -2 in BDA-labeled fibers and endings* Three vesicular glutamate transporters (VGluTs) so far have been identified in brain. These are proteins located in the walls of synaptic vesicles in glutamatergic neurons. A VGluT binds cytoplasmic glutamate, carries it across the membrane and releases it into the lumen of the vesicle (review in Liguz-Lecznar and Skangiel-Kramska 2007). VGluTs thus can be said to be in charge of payloading synaptic vesicles with neurotransmitter. In the case of VGluT1 and VGluT2, the payload exclusively is glutamate, while with VGluT3, several neurotransmitters have been associated (Liguz-Lecznar and Skangiel-Kramska, 2007). The existence of a VGluT1 or VGluT2 in an axon terminal can be exploited as it signals excitatory neurotransmission. VGluT1 has been associated with neurons involved in corticofugal projections, whereas VGluT would be present in neurons involved in subcortical connectivity (Fremeau et al. 2004). VGluT1 and VGluT2 are seldomly co-expressed by neurons. Anterograde neuroanatomical tracing combined with VGluT detection provides a two-dimensional tool (‘neurochemical fingerprinting’) through which one can study excitatory, glutamatergic projections in detail (Fig. 5a, schematic). Prerequisites for successful application of neurochemical fingerprinting are high-resolution anterograde tracing, good fixation, highly specific immunostaining, and high-resolution double-fluorescence confocal microscopy. Here, we investigated whether amygdalostriatal connectivity contains VGluT1 or VGluT2, that is, can be associated with excitatory amygdaloid action upon the striatum.

In rats, BDA was injected into nuclei of the amygdaloid complex (Fig. 6a). Following the above post-surgery procedure, sections containing BDA-labeled fibers were, after rinsing and blocking (5% donkey normal serum) steps, incubated overnight at room temperature in cocktails of two antibodies: guinea pig anti-VGluT1 and rabbit anti-VGluT2 [1:1000; next, the sections were rinsed three times in TBS-TX and incubated with a cocktail made up of streptavidin-Alexa Fluor^®^ 546 (1:400; Molecular Probes) donkey anti-rabbit-Alexa Fluor^®^ 488 and donkey anti-guinea pig-Alexa Fluor^®^ 633 (1:400; Molecular Probes)]. After rinsing the standard BDA mounting-coverslipping procedure was followed. In essence, we had three tags: VGluT1 green, BDA-labeled fiber red, and VGluT2 infrared fluorescence.

These slides were scanned in a Leica TCS-SP2 AOBS confocal laser scanning microscope equipped with 488 nm, 546 nm, and 647 nm lasers. We found extensive distribution of amygdaloid fibers in the ventral striatum in cases with BDA injection in the basomedial nucleus (example injection site in Fig. 6a). BDA-labeled amygdalostriatal fibers contained immunofluorescence signal associated mostly with VGluT1 (Fig. 6b–f, inset Fig. 6f) and, to a lesser degree, with VGluT2. Colocalization of VGluT1 and VGluT2 was extremely rare (Wouterlood et al. 2018a).

*Example D: dual-anterograde tracing with PHA-L and BDA combined with retrograde tracing with rabies virus* Here, our goal was to elucidate (glutamatergic) afferents reaching striatofugal neurons projecting to the substantia nigra pars reticulata (SNr) in rats. For this purpose, a multi-tracing paradigm was designed comprising iontophoretic delivery of PHA-L into primary motor cortex, iontophoretic delivery of BDA into the parafascicular thalamic complex, and deposit of rabies virus (pressure-injected) into the SNr. After 1 week post-surgery survival, animals were perfused and stained using a goat-anti-PHA-L antibody followed by a donkey–anti-goat Alexa Fluor^®^ 633 conjugated IgG; an Alexa Fluor^®^ 546-conjugated streptavidin (for BDA detection); and a non-commercial rabbit anti-rabies antibody followed by an Alexa Fluor^®^ 488-conjugated donkey–anti-rabbit IgG (Fig. 7). Details can be found in López et al. (2010).

##### Example E: detection of GPCR heteromers within identified projection neurons

GPCR heteromeric complexes are made of two different individual GPCRs. A GPCR complex represents a molecular entity with binding and signaling characteristics different from those of each individual (monomeric) GPCR. The recent arrival of the in situ proximity ligation assay technique (PLA; Söderberg et al. 2008) made it possible for the very first time to attempt the morphological identification of the localization of GPCR heteromers with hitherto unprecedented precision. Here, we combined PLA-based GPCR heteromer detection together with retrograde tracing with BDA. Striatal medium-sized spiny neurons in non-human primates were retrogradely labeled with BDA after tracer delivery into the internal division of the globus pallidus. Briefly, the PLA protocol was carried out first and GPCR heteromers were identified as red fluorescent spots (each one made up of combination of two different individual GPCRs). Once the PLA protocol was completed, the BDA protocol was conducted by taking full advantage of an Alexa Fluor^®^ 488-conjugated streptavidin (Fig. 8). Details can be found in Rico et al. (2017)

### Biotin derivatives and carbocyanine dyes

#### Biocytin and neurobiotin

A low-molecular weight complex of biotin conjugated to lysine named ‘biocytin’ was introduced in intracellular neurophysiological recording by Horikawa and Armstrong (1988). After finishing a recording, the biocytin was ejected from the recording pipette into the neuron with the purpose to fill the cell completely. Because this type of study investigates both functional aspects and morphology, it is safe to speak of a two-dimensional approach. Intracellular neurophysiology followed by intracellular filling with dye has a history that goes back to the initial experiments by Stretton and Kravitz (1968). King et al. (1989) reported that biocytin, after extracellular injection, appears to be taken up by small pools of neurons in the direct vicinity of the pipette tip and becomes transported mostly anterogradely. These authors reported that in cases of large mechanical injections, some injection track labeling occurred as well as retrograde transport. Biocytin can, thus, be used label to fiber projections; it has been applied for this purpose in fish species (e.g., Northcutt and Westhoff 2011).

*N*-(2-aminoethyl)biotinamide, a biotin derivative marketed by Vector (Burlingame, CA) under the brand ‘Neurobiotin’, was introduced by Kita and Armstrong (1991) as an alternative to biocytin to fill neurons with a neutral marker dye after intracellular neurophysiological recording. Neurobiotin, just like biotin and biocytin, reacts irreversibly with streptavidin and, therefore, can easily be stained with any of a color chart of fluorochrome-conjugated streptavidins. Neurobiotin has been applied mostly as an intracellularly applied marker in mammals (rat, rabbit; as exemplified recently by Ruigrok et al. 2011), amphibians (Zhang et al. 2006), and invertebrates (Fan et al. 2005). In our own research, we have used neurobiotin as a tool in a two-dimensional approach to label small populations of neurons (Fig. 3d) (Wouterlood et al. 2004). The procedure and equipment for injection of neurobiotin is similar to that for PHA-L and BDA. A special application of neurobiotin has been neuroanatomical tracing in slices of post-mortem human brain (Dai et al. 1998a, Dai et al. 1998b).

The characteristics of neurobiotin and biocytin have been extensively reviewed by Lapper and Bolam (1991) and by Smith (1992). These authors confirmed King et al.’s (1989) observation that both compounds show a tendency of being transported retrogradely. Survival times after biocytin or neurobiotin injections need to be relatively short (1–4 days), because the compounds are quickly metabolized. Both compounds are best when being used for intracellular injection and for short-distance anterograde/retrograde tracing. Another application for biocytin and neurobiotin is juxtacellular injection that results in the complete labeling of single or very small groups of neurons, including their axons (Pinault 1996). Neurobiotin or biocytin application can be made multi-dimensional by combination with electrophysiological recording (Taverna et al. 2004) and chemical phenotyping (Toney and Daws 2006). Li et al. (1990) reported successful application of biocytin as an intracellular-injection marker in neurons in slices of fixed brain. Delivering the colorless biocytin to cells in slices of lightly fixed brain can be quite challenging. Li et al. (1990) used for this purpose micropipettes loaded with biocytin mixed with the fluorescent dye, Lucifer yellow. Liu et al. (1993) introduced a colored derivative of biocytin named ‘biotin-dextran miniruby’. One spectacular study using biocytin, neurobiotin, and BDA is that exploring the trigeminal innervation of the Schnauzenorgan of the elephant nose-fish, *Gnathonemus petersii* (Amey-Özel et al. 2015).

#### Carbocyanine dyes

Application of one of the extremely lipophilic carbocyanine dyes, DiA, DiI, or DiO, results in uptake and diffusion exclusively in lipid compartments, e.g., myelin sheaths (Godement et al. 1987; Honig and Hume 1986; Mufson et al. 1990, review by Honig 1993). Other high-affinity lipophilic dyes, e.g., several ‘color’ NeuroView^®^ dyes, have been added to the repertoire (Fritzsch et al. 2005; Jensen-Smith et al. 2007). These dyes are, therefore, suitable to label myelinated fiber tracts. As active transport is not involved, the spread of the staining is bidirectional. This diffusion type of ‘tracing’ is well positioned to be used in fixed tissue, e.g., embryonic (Catalano et al. 1996; Makarenko 2007), brain of newborn mammals (cat; Gabriele et al. 2006), or in fixed, post-mortem human brain (Mufson et al. 1990; Molnar et al. 2006; Heilingoetter and Jensen (2016). Diffusion implies that the time required to label sufficient lengths of axon, is considerable. In living organisms, DiA and DiI are being used for instance in fish to track myelinated fiber projections (D’aniello et al. 2015). Axon tracing procedures in ex vivo brain material, mostly with lipophilic dyes but also including several intracellular dye injection procedures, are extensively reviewed by Heilingoetter and Jensen (2016).

### Tritiated amino acids and radioautography

A technique not much practiced any more today, yet worth mentioning, is the labeling of proteins in neurons through injections of radioactive amino acids into CNS loci. The neurons in the injection spot internalize the amino acids to incorporate them into oligopeptides and proteins which, in turn, are transported along the projection fibers towards the axon terminals. During the transport, decaying isotopes emit alpha particles that can be detected with a radioautographic detection procedure. This method was pioneered as a tracing method by Grafstein (1967) and fully developed as a tracer in the CNS by Cowan et al. (1972). Because this is a true transport-based method and because anterograde transport is exploited, the impact on neuroscience of this method has been considerable. Cowan and Cuénod (1975) and Swanson (1981) provide extensive coverage of this type of neuroanatomical tracing.

### Particles: large and small

Larry Katz ET AL. reported in 1984 that rhodamine tagged latex microspheres (diameter 20–200 nm) are internalized at axon terminals and retrogradely transported to the neuronal cell body (Katz et al. 1984). Katz’ beads can be categorized as ‘microparticles’, to distinguish them from smaller ‘nanoparticles’ (mostly metallic gold; diameter < 20 nm). Later, Katz expanded his latex bead family with green fluorescing microparticles (Katz and Iarovici 1990) to enable double-label tracing. These beads were used in connectivity studies of feline visual cortex (e.g., Katz 1987). Discrete particles offer in neuroanatomical tracing a particular advantage over macromolecules, because they make it possible without much complex chemistry to carry the study of neuronal connectivity from the light microscope level over into the electron microscope realm (Egensperger and Holländer 1988). Microparticles diffuse marginally at the site of injection; they are chemically inert and they persist for long. Katz et al. (1984) reported that microparticles have to fulfill certain physical and chemical conditions to be sufficiently attractive to neurons to become internalized. The same seems to be true for nanoparticles (Jung et al. 2006; Mendoza et al. 2010). In spite of its attractiveness, particle-based tracing never went viral: today, this type of tracing occupies its own modest niche in the universe of neuroanatomical tracing (e.g., Shafton and McAllen, 2013; Appeltants et al. 2000 and similarly, Wang et al. 2013 with 30–40 nm-diameter red fluorescent carboxylate-modified latex microspheres). Gold nanoparticles (10–12 nm) coated with lectin (Basbaum and Menetrey, 1987; Ruigrok et al. 1995; for detailed preparation of gold-lectin nanoparticles, see Ruigrok and Apps 2007) are taken up by axon terminals and retrogradely transported to the cell body where they can be made visible for light microscopic viewing through a silver enhancement step. Compatibility of lectin-coated nanogold particle tracing exists with the other neuroanatomical tracing methods (Basbaum and Menetrey 1987; Ruigrok and Apps 2007) and also with non-tracing histochemical procedures, e.g., in situ hybridization (Jongen-Rêlo and Amaral 2000). A quite recent development is to conduct retrograde fluorescent latex microparticle tracing and follow-up with intracellular injection of Lucifer Yellow (Vercelli et al. 2000) or diolistic labeling with DiI of target neurons whose cell bodies contain transported microparticles (Neely et al. 2009).

In the peripheral nervous system, Cholera toxin B conjugated carbon dots have been used to retrogradely trace connectivity (Zhou et al. 2015).

A different application of nanoparticles is to use them as non-cytotoxic vehicles to deliver viral vectors into neurons (Bharali et al. 2005; 30 nm size silica particles). The nanoparticles serve two roles here: carrying the tracing flag while at the same time delivering a payload such as a drug or a photosensitive dye for photodynamic cancer treatment (Roy et al. 2003).

In fact, the Bharali et al. (2005) experiment bridges the gap between inorganic nanoparticle tracing and tracing with ‘natural’ nanoparticles, i.e., viruses (dimensions measured in angstroms and molecular mass determined in daltons). As the increasingly ‘popular’ adenovirus capsid measures approximately 90 nm across (900 Å; Harrison 2010) this agent can be considered as a small, hollow organic microparticle that can be tweaked to carry a payload. Contrary to an inorganic particle that must be coated with an appealing agent to make it sufficiently attractive for a neuron to ingest it, a virus is much more effective. It possesses the receptor complements to attach to neurons of choice and inject them with DNA or RNA fragments to force their molecular machinery to start producing an endless stream of duplicates of the virus or a particular metabolic product. We will return to this issue later in this review.

### Non-fluorescent and fluorescent retrograde tracing

#### Good old horseradish peroxidase

The ‘archmother’ of the neuroanatomical transport tracers is without doubt the enzyme, horseradish peroxidase (HRP). The first studies on retrograde transport of HRP were one-dimensional (Kristensson and Olsson (1971a,b), wherein these researchers reported that they had observed labeling of spinal motoneurons in rats after deposition of HRP in the gastrocnemius muscle. Detection was done colorimetrically with diaminobenzidine (DAB) substrate (Graham and Karnovsky 1965). In another report in 1971, Kristensson and Olsson (1971a) described in rats labeling of motoneurons in the hypoglossal nucleus following injection of HRP into the tongue. The first report of tracing central connectivity in brain followed in the next year (LaVail and LaVail 1972). Today, HRP is considered to be a bidirectional tracer with asymmetry in transport direction (i.e., overwhelmingly retrograde). The most common types of HRP used for tracing are Sigma Type VI (Sigma-Aldrich/Merck, St. Louis, MO) and Boehringer (Boehringer–Ingelheim). The enzyme is applied in solution by mechanical injection, or applied as a foam gel, crystals or pellets, and it is subsequently internalized by fluid-phase endocytosis into membrane clad endosomes. In the interior of the neuron, the HRP remains endosome packaged. The endosomes are transported towards the perikaryon where they fuse with lysosomes to undergo degradation by endogenous peroxidase.

A long list can be made up in the literature of connectivity studies using HRP as the tracer of choice; HRP is still being used (Afarinesh and Behzadi, 2017). In the course of only one decade following the publications by Kristensson and Olsson, the HRP tracing technique matured; most of what is necessary to conduct successful HRP tracing can be found in reference works such as the books by Mesulam (1982) and Bolam (1992). A protocol type of discussion of HRP tracing, including all the chromogens available for light and electron microscopy, is provided by Mesulam and Rosene (1979) and, more recently, by van der Want et al. (1997). In standard HRP tracing, the contrast between the formed precipitate versus background can be enhanced by the addition of nickel–ammonium sulfate to the incubation medium (Ni enhancement; Hancock 1986).

##### Fluorescent retrograde tracers

While tracing with HRP requires delicate histochemical processing, a faster, more ‘no non-sense’ way of visualizing neuronal connectivity would be via the uptake and transport of a tracer that fluoresces by itself. This simple idea had been addressed by Kristensson 1 year before his landmark introduction of HRP. Thus, in 1970, Kristensson reported on an experiment in which he had injected a conjugate of albumin and the fluorescent dye, Evans Blue into the gastrocnemius muscle of rats. After a post-surgical survival period, fluorescence signal was detected in spinal motoneurons (Kristensson 1970). Maybe the idea was put in a lab drawer by the gigantic success of the HRP story 1 year later, because it took nearly a decade before Kristensson’s idea was repeated, this time with inorganic fluorescent dyes injected into the CNS (Bentivoglio et al. 1979; Kuypers et al. 1979). Similarly to HRP, the inorganic fluorescent dyes appear to be taken up by nerve terminals and transported retrogradely to their parent cell bodies. A repertoire of retrogradely transported fluorescent dyes has since been introduced: bisbenzimide, DAPI primuline, propidium iodide (Kuypers et al. 1979), True Blue, granular blue (Bentivoglio et al. 1979), nuclear yellow, Fast Blue (Kuypers et al. 1980; Bentivoglio et al. 1980), and diamidino yellow (Keizer et al. 1983). Of these, Fast Blue and diamidino yellow are still being applied frequently. Depending on their composition, retrogradely fluorescent dyes may accumulate in the perikaryal cytoplasm, bind to nuclear components such as nucleic acids, or may accumulate in specific cell organelles. For instance, Fast Blue accumulates in the cytoplasmic compartment, whereas diamidino yellow lights up nuclei. While the early fluorescent tracers suffered from photobleaching under UV illumination, a newer generation of retrograde fluorescent tracers such as Fluoro-Gold (FG; hydroxystilbamidine; accumulates in the cytoplasmic compartment) (Schmued and Fallon 1986) and Fluoro-Ruby (rhodamine-dextran-amine; Schmued et al. 1990) appears very robust as well as extremely photobleaching resistant. In a living animal, FG may resist metabolic breakdown up to a year post-injection. Its intense and bleach-resistant labeling has contributed to raising FG to the level of ‘gold standard’ for fluorescent retrograde labeling in rodents, particularly for multiple labeling in combination with other tracers (Fig. 5, schematically) (see Abdel-Majid et al. 2005; Wouterlood et al. 2018b). Fast Blue is being phased out after the initial manufacturer (Dr. Ihling, Germany) ceased production; currently, this compound is back in the catalog of the Sigma-Aldrich company. Nevertheless, Fast Blue in combination with diamidino yellow still is being used in double-retrograde fluorescent labeling studies.

While FG is known as a retrograde tracer per se, Fluoro-Ruby is also transported anterogradely (Bowyer and Schmued 2006), as it is chemically related to BDA.

Fluorescent tracers (retrograde as well as anterograde) offer several characteristics that are very useful for neuroanatomical connectivity research. First, the label is directly visible in a fluorescence microscope without any histochemical or immunohistochemical treatment. Second, many of these tracers (e.g., FG) can be used to trace extremely long fiber tracts, e.g., corticospinal and bulbospinal projections (e.g. Rice et al. 2010). Special characteristics of FG are that it survives rigorous immunofluorescence procedures and that it can be used to mark neuronal cell bodies as targets for intracellular injection post fixation (Abdel-Majid et al. 2005; Wouterlood et al. 2018b). Finally, because of their spectral characteristics, fluorescent retrograde markers serve as near-ideal tracers to study axon collateralization in double-labeling experiments and, in a multi-dimensional setting in combination with immunofluorescence, to study the neurochemical identity of traced fiber projections (an approach pioneered by Van der Kooy and Steinbusch 1980; reviewed by Huisman et al. 1984 and by Wessendorf 1990). Successful application of quadruple retrograde fluorescence tracing to follow medium and long tracts in the CNS of rats was conducted by Akintunde and Buxton (1992), whereas Richmond et al. (1994) systematically compared results with seven different retrograde tracers in a cat peripheral motor nerve model: Fast Blue, FG, fluorescein-dextran, rhodamine-dextran-amine (RDA), fluorescent latex microspheres, HRP, and DiI. Schofield et al. (2007) reported different results with mixtures of two retrograde fluorescent tracers injected into one spot in the CNS. An interesting observation is that retrogradely labeled ganglion cells in rat retina may pass FG via gap junctions to amacrine cells (Abdel-Majid et al. 2005).

Application of all these tracers is mostly via mechanical injection with a Hamilton syringe or glass micropipette. A few studies report microiontophoretical delivery (e.g., Schmued and Heimer 1990). Most fluorescent tracers can be dissolved in water. Diamidino yellow is difficult to dissolve and is typically suspended at 2–3% in 200-mM phosphate buffer, pH 7.2 (Keizer et al. 1983) and sonicated just before use. Diamidino yellow produces smaller injection sites than Fast Blue and FG, which may be an advantage depending on the system that is being investigated. Transport times for long distance fiber connections in primates which range between 1 and 5 weeks, depending on the distance involved (e.g., 5 weeks for strong labeling of corticospinal and corticobulbar neurons in macaque monkeys after injection of diamidino yellow into the cervical spinal cord and injection of Fast Blue into the lower medulla; Keizer and Kuypers, 1989). After the required post-surgery transport period, brains are commonly fixed via perfusion–fixation with phosphate-buffered 4% depolymerized paraformaldehyde. A trace of glutaraldehyde (0.05%) is allowed to improve fixation, although glutaraldehyde strongly contributes to ‘green’ fluorescence background signal. After fixation, the tissue can be cut immediately on a vibrating microtome, or cryoprotected, frozen, and then cut at 40–60-µm thickness on a freezing microtome.

#### Fluoro-Gold: application notes and source

FG can be considered as today’s flagship of the retrograde fluorescence tracer repertoire (Schmued 2016), especially in multi-dimensional tracing procedures in which the second and/or third layers are immunofluorescence procedures. One disadvantage of FG in the latter type of studies is that its emission spectrum is quite wide. FG-related emission thus may cross bleed into the channel reserved for the second or even third fluorescent label. In confocal laser scanning studies, FG is less favored because of this, but also because its excitation peak sits at 323 nm (Schmued and Fallon, 1986) which requires an extra (expensive) laser. This disadvantage can be circumvented by not relying on FG itself but instead on an anti-FG antibody (Chang et al. 1990). Antibodies against FG have been successfully used as well to carry over light microscopical tracing to the electron microscope (Deller et al. 2000).

FG is marketed by Fluorochrome, Inc. (Denver, CO), and it is applied through mechanical injection or iontophoresis. It can be dissolved in a range of buffers (e.g., 5-mM acetate buffer, pH 5.0, 100-mM phosphate buffer, pH 7.4) of which 100-mM cacodylate buffer pH 7.3 seems favorite for either a mechanical application via a Hamilton syringe or via microiontophoresis (Schmued and Heimer, 1990). Survival periods range from 1 week to 1 year.

##### An impressive track record: the CTB family

A great step forward in the evolution of HRP tracing was conjugation of native HRP with the (non-toxic) B subunit of cholera toxin (CTB-HRP) or with wheat germ agglutinin (WGA-HRP) (Trojanowski et al. (1981). Tracing with these conjugates 60-fold improved the sensitivity of the tracing method compared with native HRP, while an anterograde transport component of the tracer became evident. The strongly enhanced sensitivity hinges on three features. First, the uptake of CTB-HRP or WGA-HRP by neurons occurs via a receptor-mediated uptake mechanism called adsorptive endocytosis, while native HRP is taken up by less-efficient fluid-phase endocytosis (reviewed by Trojanowski 1983). Second, detection of transported CTB-HRP and WGA-HRP requires immunohistochemistry which is much more sensitive than the ‘plain’ colorimetric histochemistry used in the classical HRP procedure. Third, the immunohistochemical reaction itself was markedly improved by the introduction of a new chromogen: tetramethylbenzidine instead of diaminobenzidine (see Mesulam 1982).

CTB-HRP has been reported to become less rapidly eliminated from retrogradely labeled neurons than native HRP (Wan et al. 1982), probably because the CTB-HRP is transported along a different intracellular pathway than native HRP (Trojanowski, 1983). Thus, both CTB-HRP and WGA-HRP can be regarded as bidirectional tracers. Anterograde axonal transport occurs at a fast rate (e.g., about 108 mm/day for WGA-HRP); native HRP is anterogradely transported slower, at a rate of 288–432 mm/day (Trojanowski 1983).

Lindh et al. (1989) and Luppi et al. (1990) were the first to go one step beyond Trojanowski et al. by conducting tracing with CTB in its unconjugated (‘plain’) form. A big advantage compared with HRP and CTB-HRP is that ‘plain’ CTB is exclusively transported retrogradely. Another advantage of ‘plain’ CTB is that its detection does not interfere with intrinsic peroxidase activity present in peroxisomes and lysosomes. One of the big concerns in classical HRP tracing was rapid development of background staining during the histochemistry (Mesulam 1982). Such background staining is in principle absent when ‘plain’ CTB is used in combination with immunohistochemical detection. ‘Plain’ CTB, followed up by immunofluorescence detection, is, therefore, the (non-fluorescent) retrograde tracer of choice today. Direct CTB tracing with rhodamine- or fluorescein-conjugated CTBs has been performed by Lyckman et al. (2005) in the visual system of mice. A series of robust, photostable Alexa Fluor^®^ conjugated CTB molecules is nowadays available (Conte et al. 2009a, b). Just like the inorganic retrograde fluorescent tracers, these compounds are directly visible in a fluorescence microscope. Because CTB is considered a retrograde tracer *pur sang* and also because it has proven its compatibility with other neuroanatomical tracing regimes, it is in double-immunoperoxidase and immunofluorescence conditions often applied in multi-dimensional studies, e.g., in combination with the anterograde tracer PHA-L (Berendse et al. 1992b), BDA (Atoji et al. 2018; Arima et al. 2019), combined with standard immunofluorescence procedures to either visualize the chemocytoarchitectonics of the area (Berendse et al. 1992a) or to chemically phenotype the retrogradely labeled neurons (Gumbs et al. 2019) (schematic, Fig. 5b). CTB can be used in a double-retrograde tracing experiment with Fluoro-Gold; in one of such labeling experiments, the tracers were injected each on a different side of the midline in the superior colliculus in rats (Yao et al. 2018). Minor differences were reported in retrograde staining of neurons in the retina. Finally, double direct retrograde tracing using two different fluorescence-conjugated CTBs has been reported in the PNS (Maeda et al. 2017). The far majority of CTB tracing studies have been conducted in a variety of mammals, while reports on work in pigeon (e.g., Atoji and Wild 2004; Atoji et al. 2018) provide evidence that this tracer also can be applied in non-mammals.

Bacterial toxins other than CTB have been proposed but have never become so dominating the scene as CTB. For instance, the (non-toxic) C fragment of tetanus toxin (TTC) conjugated to Lac-Z (Coen et al. 1997) or to GFP (Maskos et al. 2002) as (transsynaptic) retrograde tracers have been reported.

## The slow, unstoppable rise of viruses as tracing tools

The exploitation of viruses has an exciting history. Initially, viruses were introduced as nanoparticles whose presence and travel routes are traceable; today, they are increasingly used as delivery systems for genetic payloads that subsequently turn neurons into tracer–producing molecular machines. In human medicine, the fog of history shrouds the origin of the awareness that a mysterious agent, transmitted via kissing or sexual contact, causes blisters on the lips, in the oral region or genitals. Hippocrates and the emperors of Rome seemed to be aware of this phenomenon, and Shakespeare is said to have mentioned it in his ‘Romeo and Juliet’. In the 1920s, the vector of the disease was identified as Herpes simplex virus (HSV). Type-1 HSV-1 infects skin epithelial cells, multiplies, migrates to nerve endings and then travels along sensory nerves to settle in neuronal perikarya inside spinal and cranial nerve ganglia (Goodpasture and Teague 1923; Johnson 1964). The presence of HSV nanoparticles inside axons was confirmed in 1972 by electron microscopy (Hill et al. 1972).

### First-generation vector: neurotropic viruses

These early observations prompted researchers in the 1970s to test the usefulness of HSV in tracing paradigms in the CNS and PNS (Kristensson et al. 1974, 1982; Bak et al. 1977). Ugolini et al. (Ugolini et al. 1987, 1989) recognized the ‘natural’ signal amplification by virtue of virus replicating itself, and they praised the unique potential of virus particles to cross synapses to infect second-in-line neurons (Fig. 9). The latter feature produces labeled neurons that in neuronal networks synaptically communicate with the infected neuron (Ugolini et al. 1989). Initial problems were the runaway nature of retrograde viral infections and the highly virulent appetite of native HSV-1 for humans which required rigorous laboratory biosafety containment measures. HSV-1 appears to be transported bidirectionally in an asymmetrical way, that is, strongest retrogradely (Ugolini 2010). A search for a virus propagates exclusively in the retrograde direction brought (canine) rabies virus (RV, Ugolini 1995) (review: Junyent and Kremer 2015) and swine rabies virus (PRV) (pseudorabies virus; Martin and Dolivo 1983; Loewy 1995; review: Callaway 2008) into the spotlight. Once receptor-mediated fusion of a rabies virus envelope with the axon terminal’s membrane has succeeded and the capsid has become internalized, this material becomes attached to rapid, dynein mediated fast (retrograde) axonal transport along microtubules. Once arrived in the cell’s perikaryon the viral DNA strands target the nucleus (Sodeik 2000; Pomeranz et al. 2005; Ugolini 2010). Today, attenuated rabies virus strains such as CVS-11 (Ugolini 1995) PRV (Jansen et al. 1995)/PRV–Bartha (Aston-Jones and Card 2000) and CVS-N2c^Δ*G*^ (Reardon et al. 2016; Zhu et al. 2019) provide reliable tools for retrograde tracing of neuronal connectivity with the added value of transsynaptic labeling. Recently a safe, neuron-friendly double-deletion RV vector was introduced (Chatterjee et al. 2018).

Apart from the above viral strains, ‘plain’ RV exhibits strong neurotropism and transsynaptic passage (reviewed in Kelly and Strick 2004), therefore, appointing this virus as an excellent tool for elucidating neuronal ensembles, such as those giving rise to cortico-striato-nigral and cortico-striato-pallidal pathways (Fig. 9) (Deng et al. 2015). RV is an RNA virus that exhibits a higher efficiency for retrograde transport than HSV together with a lower cytopathogenicity (Ugolini 1995). Although RV can be detected with antibodies recognizing a viral nucleoprotein, thus resulting in labeling of neuronal soma and main thickest dendrites, the thinnest dendritic processes and dendritic spines cannot be detected through this approach. This limitation has been circumvented by performing viral detection with antibodies directed against a soluble viral phosphoprotein (31G10; isolated by Raux et al. 1997) that spreads throughout the cytoplasm of RV-infected neurons. The result is Golgi-silver staining-like retrograde labeling of these neurons (see Fig. 7) (Salin et al. 2008, 2009; López et al. 2010).

### Recombinant viruses that transfect cells to produce a fluorescent marker

At the end of the twentieth century, the synergy between molecular biology and virology reached the point that viral vectors could be seemingly at will constructed in virology labs around the world. A remarkable, novel feature of virus application was touched at the end of the previous section: the ability to switch on gene expression in infected cells. This feature is increasingly being exploited by virus-based tracing.

One of the first reports using a recombinant virus primarily to act as a delivery agent to transfect neurons in the CNS with a gene that codes for GFP was by Chamberlin et al. (1998) who used an adeno-associated virus (AAV). Here, an anterograde tracing paradigm was established, because in the transfected neurons, fluorescent protein accumulates everywhere, most importantly in the axon down to the axon terminals. In the same time frame, the unraveling of the molecular structure of viruses (PRV: Pomeranz et al. 2005; AV: Fuschiotti et al. 2006; Harrison 2010) made it possible to proceed even further. An explosion followed of combinations of preferred features taken from different, useful viruses and assembled into new, unique and highly specific vectors. The insertion of a gene coding for lacZ or GFP in a PRV-Bartha strain made multiple-synapse retrograde tracing more efficient (Boldogköi et al. 2002). This PRV-152 ‘conjugate’ was subsequently successfully applied as transsynaptic tracer in the rat ortho- and parasympathetic systems (Szabó et al. 2015; Ahn et al. 2018). A similar GFP Cre-dependent PRV vector approach has been used to trace connectivity in the basal ganglia (Ribeiro et al. 2019). Runaway second-order and higher order virus infection, however, remains a danger. As glycoprotein G appears to be a key ingredient in multisynaptic retrograde transfer of virus (Mazarakis et al. 2001), a rabies virus deficient for glycoprotein G (Mebatsion and Gonzelmann 1996; reviewed in Callaway, 2008) was proposed as a tool for the first-order-neuron-only retrograde tracing tool (as illustrated in Fig. 7); the subsequent substitution in Wickersham’s lab of the gene coding for glycoprotein G by one coding for EGFP produced a powerful, first-in-line-neuron-only retrogradely transported vector that, by virtue of expressing EGFP in infected cells, made retrogradely labeled, infected neurons directly visible under fluorescence (SADΔG-EGFP; Wickersham et al. 2007a; Kim et al. 2016). High specificity combined with a low biosafety hazard level has been obtained with a G protein-deleted PRV strain SAD B19 with a GFP cassette included in their genome. These viruses lack glycoprotein necessary for infection; virus infection is started by means of a second injection of a ‘helper’ virus that switches on GFP expression by the infected neurons (review in Ghanem and Conzelmann, 2016). Some classes of neuron seem to resist SADΔG-eGFP and similar fluorescence transfecting vectors (Albisetti et al. 2017). The synaptic specificity in transsynaptic virus tracing is not yet completely understood and needs to be further investigated (Beier, 2019).

While most attention focused on canine and swine rabies virus, ‘good old’ HSV-1, i.e., the strain H129 ΔTk-TT, received renewed attention as a tool to trace transsynaptically in an anterograde fashion, while carrying a Cre-dependent loxP-STOP-loxP-tdTomato-2A-TK cassette (Lo and Anderson, 2011), making this tool only usable in transgenic mice.

Up to this stage, most publications on virus tracing represent the ‘first generation’ of application; viruses used primarily as a one-dimensional tracing tool with the purpose to chart the retrograde spread of infection along pathways and synaptic junctions into second and third order of neurons in the PNS and CNS. Also, most work was done in rats and mice; to have a more universal retrograde transsynaptic virus tracing instrument at hand, Beier et al. (2017) tested recombinant tracing with vesicular stomatitis virus (rVSV) in a range of animal species, e.g., chicken and zebrafish. Recently, Li et al. reported an attempt to improve retrograde neurotropic virus tracing that included a canine adenovirus 2 (CAV-2) vector constructed to force neurons to express adenovirus receptors on their outer membranes (Li et al. 2018).

#### Sidestep into the world of adenoviruses

A short diversion into adenoviruses (AV) seems appropriate here. Adenoviruses are medium-sized (90–100 nm), icosahedral entities composed of a nucleocapsid and a double-stranded linear DNA genome (Harrison, 2010). Upon infection, only the viral DNA enters the host cell and is immediately transcribed. Because this DNA is not incorporated into the host’s genes, it is not replicated when cell division occurs, and for that matter, the virus DNA does not occur in descendants of the infected host cells. The used adenoviruses are non-toxic and do not offer a biohazard to humans. These characteristics render AV and adeno-associated viruses (AAV) perfect candidates to engineer into instruments to functionally interrogate neural circuits (review by Sun and Schaffer, 2018), for instance to introduce a ‘light switch flipping gene’ into a neuron.

### Viruses flipping the light switch in specific neurons

At this point, the term ‘vector’ should be precisely defined. Nassi et al. (2015) provide such a definition: a derivative of a virus used for the delivery of genes. As gene transfer into host neurons is necessary to promote replication of virus particles, e.g., in a simple tracing paradigm or in attempts to flip gene expression on, deliver genetic payload or to completely overhaul the host’s metabolism, we will further use in this review the term ‘viral vector’. Besides providing an operational definition of ‘vector’, Nassi et al. (2015) document all current viruses of interest to neuroscientists (Table 2 in Nassi et al. 2015).

Here, the onset of a new generation, i.e., virus-vector tracing, can be distinguished, in which the emphasis of the application is not so much tracing connectivity as well as the delivery of a payload targeting the host neuron’s genome. This payload may be a gene or a drug. One important further step in the evolution of the virus-vector tracing techniques was to increase the specificity of the tracing system. For this purpose, several solutions have been devised. We identify a few here.

#### Microcircuit tracing with genetically altered rabies virus in Cre mice

Microcircuits are neuronal ensembles consisting in their most simple form of a ‘main neuron’ surrounded by interneurons impinging on it through synaptic contacts. The projection neurons in nuclei’A’ and ‘B’ in Fig. 1 can be considered as ‘main neurons’ in microcircuitry terminology. Standard neuroanatomical tracing methods are poorly equipped to study microcircuits, because the brain volume occupied by a microcircuit is in the same order of magnitude as an injection spot produced with a classical neuroanatomical tracer (Fig. 2a). With anterograde tracers, microcircuits usually remain obscured by the entanglement of labeled perikarya, dendrites, and fibers in the injection spot. Application of a retrograde tracer does not help, because these tracers only label the projection neurons (Fig. 2b). Up to now, basically two instruments were available to study microcircuits: intracellular neurophysiology (e.g., Sik et al. 1993; Klausberger and Somogyi 2008) and Golgi-silver impregnation (e.g., Bolam and Ingham 1990).

In 1998, Chamberlin et al. pioneered on a novel, third instrument: genetically engineered viruses. Creative engineering with rabies virus that followed plus the availability of genetically engineered mice has perfected this third instrument: tracing with pseudotyped G-deleted rabies virus in Cre-dependent TVA mice, as introduced by Wickersham et al. (Wickersham et al. 2007b; Callaway and Luo 2015) (Fig. 10a), expanded by Osakada et al. (2011), and beautifully conducted by Oyibo et al. (2014), Sun et al. (2014), and Zingg et al. 2018. Here, two injections of vectors into the brain are necessary: the first one is with a non-replicating AAV LoxP-Lox2272 helper virus which activates the expression of GFP and B19-glycoprotein through Cre. This injection creates a spot containing GFP ‘starter’ neurons (the ‘main neurons’ in microcircuits) that are rabies infection prone, because they express B19-glycoprotein on their outer cellular membranes. The second injection (in the same location) is the actual tracing step with pseudotyped G-deleted, mCherry rabies virus. Only B-19 expressing neurons (which by virtue contain also GFP) internalize the rabies vector and transfer the rabies vector via synapses to the second-in-line neurons that provide afferents to the B19-GFP neurons. The rabies vector infected cells accumulate mCherry and under appropriate fluorescence excitation will light up red, while the GFP expressing ‘main’ neurons will light up green (schematically: Fig. 10b) (explained in Callaway and Luo 2015). Sun et al. (2014) and Hafner et al. (2019) beautifully illustrate microcircuit visualization in cerebral cortex. Also at the thalamic–hypothalamic level, this approach works (Broms et al. 2017).

#### Flipping light switches: AAV and neurotransmitter-specific tracing

Classical tracing methods can be regarded as poor instruments to study connectivity of neurotransmitter-specific neurons, because inorganic tracers, lectin tracers, and ‘classical’ virus tracers in essence act non-selective. They simply go at work with all the neurons available in an injection spot. In cerebral cortex for example, deposition loci of PHA-L or BDA are usually full of labeled neurons of all sorts of pyramidal cells (that are known to be excitatory) and stellate-type neurons (most of which belong to some class of inhibitory interneuron). A consequence of this apparent lack of specificity is that one can expect in ‘chemical fingerprinting’ studies rather disappointingly low numbers of tracer-labeled axon terminals that express the second, sought-after marker (as we experienced; Wouterlood et al. 2018a). In this respect, a ‘chemical fingerprinting’ study resembles interrogating the entire population of a town to find one lone burglar. The same can be said of retrograde tracing combined with neurotransmitter immunohistochemistry with the purpose to study neurotransmitter-specific projections. Efficiency would jump to a higher order of magnitude if only cells expressing a particular neurotransmitter would become labeled, e.g., GABAergic neurons, cholinergic neurons, monoaminergic neurons, and the like. Genetic engineering and virus-vector delivery has made this possible.

Around the arrival of the twenty-first century, the first reports were published of work with strains of genetically modified mice in which specific subsets of CNS neurons express green fluorescent protein (GFP) (Sauer 1998; Callahan et al. 1998): for instance GFP expressing olfactory bulb granule cells (van den Pol and Ghosh 1998) and thy1 gene expressing neurons (Feng et al. 2000; Porrero et al. 2010). Next came neurotransmitter-specific transgenic mice with Cre-recombinase expression in dopaminergic neurons (Zhao et al. 2004; Bäckman et al. 2006; Witten et al. 2011—in rats), GAD67 neurons (Tamamaki et al. 2003), 5HT (serotonergic) neurons (Zhuang et al. 2005), choline acetyltransferase (cholinergic) neurons (von Engelhardt et al. 2007), and parvalbumin (GABAergic) neurons (Tanahira et al. 2009). A whole series of transgenic mice is currently available wherein neurotransmitter-specific neurons express fluorescent proteins of various spectral variants (Taniguchi et al. 2011). In spite of this impressive progress, one drawback remained, notably that in these mice, all cells with the corresponding neurochemical identity express some fluorescent protein. The same happens in the multispectral brainbow mice (Weissman et al. 2011). To trace axonal connectivity effectively preferably, a small portion of the neurotransmitter-specific neurons in these transgenic mice should start expressing fluorescent protein. The key vector to achieve this is an adenovirus- or adeno-associated virus vector whose primary role is not so much to trace neuronal connectivity, but, instead, to deliver a gene coding for one of the fluorescent proteins. Expression of channelrhodopsin-mCherry can be forced in Cre-recombinase mice through focal injection with a viral vector (e.g., rAAV–FLEX–rev-ChR2mCherry; Atasoy et al. 2008). Channelrhodopsin expression is mainly used in photostimulation studies. Although this fluorescent protein is applicable in tracing studies concentrations in long axons remain low with, accordingly, weak staining (Atasoy et al. 2008). For anatomical purposes, GFP and eYFP have much better properties than channelrhodopsin, because these fluorescent proteins accumulate in axons to levels sufficiently high to render fibers with all their details visible in routine and confocal fluorescence microscopy.

**Fig. 12:**
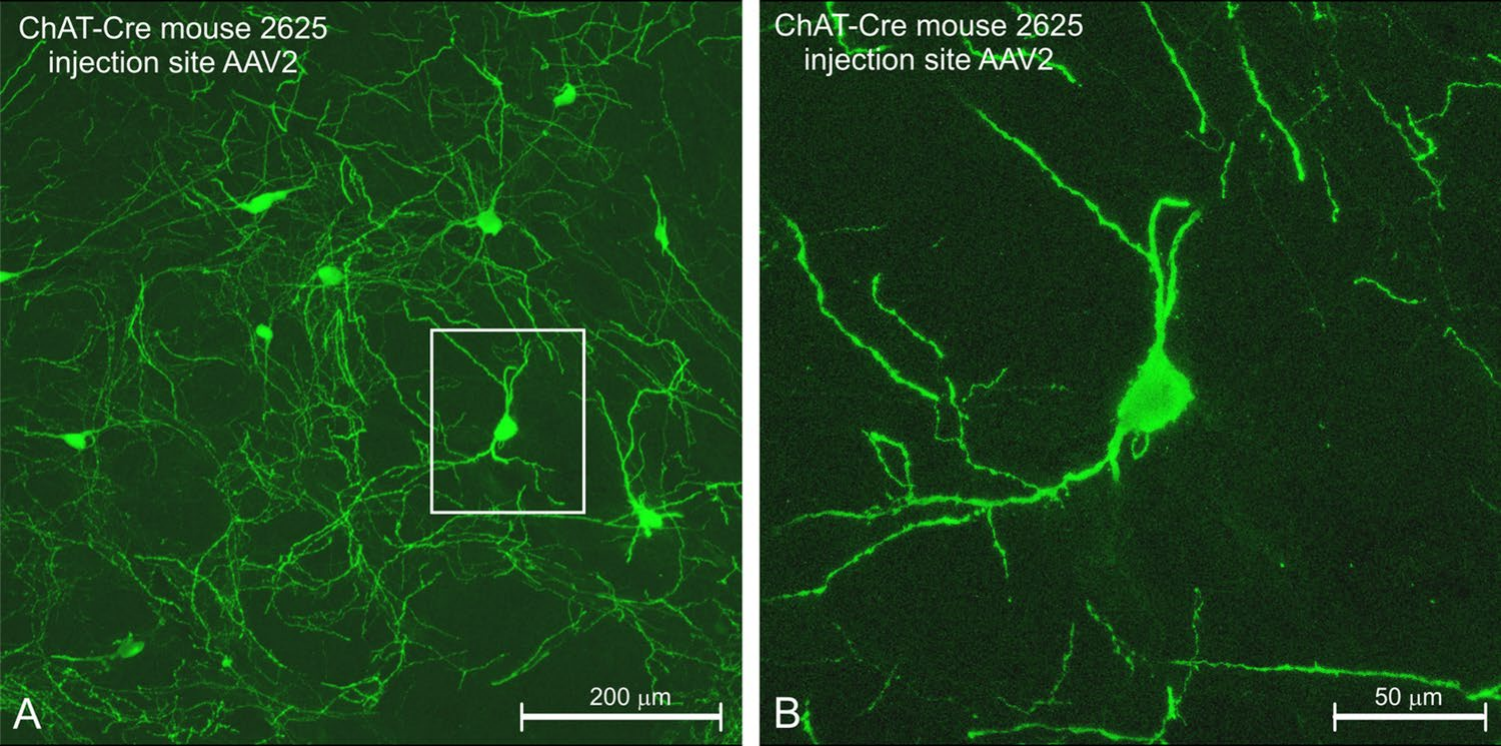
Example of tracing in a genetically engineered mouse ChAT-Cre. Expression of eYFP in cholinergic neurons, switched on through transfection after injection of a modified, double-floxed EF1α–DIO–EYFP–WPRE–pA AAV2 vector (as schematically explained in Fig. 10). **a** Confocal laser scanning Image at low magnification of an injection spot in the diencephalon–mesencephalon transition (entopeduncular nucleus). **b** eYFP expressing cholinergic neuron in the boxed area of A scanned at higher magnification. All morphological details of the neuron can be appreciated

Kuhlman and Huang (2008) experimented in this respect with AAV in Cre-recombinase knock-in parvalbumin (PV) transgenic mice. The AAV harbored a loxP–STOP–loxP cassette in between a promoter and a DNA sequence coding for a fluorescent protein, in this experiment eYFP. The trick here is that the Cre-recombinase gene occurs in the cell’s genome immediately after the gene coding for PV. As a consequence, Cre recombinase is only expressed in neurons that express also PV and only in these specific cells recombines the payload delivered by the adeno-associated virus. After AAV injection, PV neurons in the injection site promptly started to express and accumulate eYFP (Kuhlman and Huang 2008). Harris et al. (2012) further explored this idea, whereas Bloem et al. (2014) similarly used a series of topographically organized injections of an AAV-2 vector carrying the double-floxed gene, EF1α–DIO–EYFP–WPRE–pA, to map cortical projections from small subpopulations of cholinergic neurons in the forebrain cholinergic system of Cre-recombinase knock-in choline acetylcholinesterase transgenic mice (Fig. 12). Because the vector forces transfected cholinergic neurons to express eYFP (Sohal et al.; 2009; see also Madisen et al. 2010), the tracing can be considered as passive by accumulation of eYFP in all processes belonging to transfected neurons (Fig. 11c). This technique truly compares with flipping a light switch in only a few chemophenotypically specific neurons, resembling the illumination of a few individuals of a tree population in an otherwise completely dark forest (Fig. 12).

It is without doubt that AAVs collectively represent invaluable tools for a wide range of CNS applications; these include key aspects like circuit tracing, modeling neurodegenerative proteinopathies, as well as a number of therapeutic approaches under the current development (reviewed in Pignataro et al. 2018). Although different AAV serotypes exhibit some degree of retrograde transport, a number of capsid modifications have recently been designed in an attempt to boost retrograde transduction. In this regard, capsid variants such as AAV2-retro (Tervo et al. 2016), AAV-TT (Tordo et al. 2018) and AAV2-HBKO (Naidoo et al. 2018) hold great promise, allowing widespread CNS transgene expression by taking advantage of brain circuits innervating the area of deposit. Accordingly, intraparenchymal delivery of the above viral vectors resulted in retrograde spread of the transgene throughout a number of brain areas innervating the injected site. Although AAV2-HBKO has been successfully tested in non-human primates after intraparenchymal injection in the thalamus (Naidoo et al. 2018), it is also worth noting that the performance of AAV2-retro and AAV-TT capsid variants so far has only been tested in the CNS of rodents, both “retroAAVs” being injected into either the striatum (AAV2-reto and AAV-TT) or in pontine nuclei (AAV2-retro). These two brain areas host neurons but also fibers of passage coursing downstream towards their terminal endings. Uptake of the injected “retroAAVs” through fibers of passage can, therefore, not be ruled out. Overall, although the rationale for “multiple CNS areas being transduced with just one injection” looks extremely appealing, results with AAV2-retro and AAV-TT remain to be validated in non-human primates by injecting these AAV capsid variants in brain territories lacking fibers of passage.

#### Final notes

Multi-dimensional approaches in neuroanatomy have in the past 40 years replaced the pioneering, one-dimensional techniques. After a fledgling start in the 1970s, the future seems very bright for tracing studies with viruses that bring or trigger fluorescent protein expression in first- and second-in-line neurons. Viruses offer a spectacular third instrument in research aimed at elucidating the organization of microcircuits.

It should be noted, though, that viruses are specialized agents that have evolved to target specific animal species. Most, if not all, axon tracing work with viruses has been conducted with a limited range of mammalian species. In spite of this limitation viruses remain currently in the spotlight because of their incredible potential as molecular-genetic tools, not only in neuroscience (Luo et al. 2018), but everywhere in biomedical applications. One, ironic example is the approach to fight Herpes virus infections with gene therapy brought about by virus vectors (Chen et al. 2018). In human medicine, first successes have been reported using viruses in gene therapy models. Viruses may also be applied as vehicle to carry therapeutic agents to diseased neurons in neurodegenerative diseases (Géral et al. 2013) or assist in brain repair (Chen et al. 2019).
